# Four MicroRNAs Promote Prostate Cell Proliferation with Regulation of PTEN and Its Downstream Signals *In Vitro*

**DOI:** 10.1371/journal.pone.0075885

**Published:** 2013-09-30

**Authors:** Ling Tian, Yu-xiang Fang, Jing-lun Xue, Jin-zhong Chen

**Affiliations:** 1 State Key Laboratory of Genetic Engineering and Institute of Genetics, School of Life Sciences, Fudan University, Shanghai, China; 2 Experimental Research Center and Cancer Center, Shanghai First People’s Hospital, Shanghai Jiaotong University School of Medicine, Shanghai, China; 3 Stem Cell Research Center, Renji Hospital, Shanghai Jiaotong University School of Medicine, Shanghai, China; French National Center for Scientific Research - Institut de biologie moléculaire et cellulaire, France

## Abstract

**Background:**

Phosphatase and tensin homologue (PTEN), as a tumor suppressor, plays vital roles in tumorigenesis and progression of prostate cancer. However, the mechanisms of PTEN regulation still need further investigation. We here report that a combination of four microRNAs (miR-19b, miR-23b, miR-26a and miR-92a) promotes prostate cell proliferation by regulating PTEN and its downstream signals *in* *vitro*.

**Methodology/Principal Findings:**

We found that the four microRNAs (miRNAs) could effectively suppress PTEN expression by directly interacting with its 3’ UTR in prostate epithelial and cancer cells. Under-expression of the four miRNAs by antisense neutralization up-regulates PTEN expression, while overexpression of the four miRNAs accelerates epithelial and prostate cancer cell proliferation. Furthermore, the expression of the four miRNAs could, singly or jointly, alter the expression of the key components in the phosphoinositide 3-kinase (PI3K)/Akt pathway, including PIK3CA, PIK3CD, PIK3R1 and Akt, along with their downstream signal, cyclin D1.

**Conclusions:**

These results suggested that the four miRNAs could promote prostate cancer cell proliferation by co-regulating the expression of PTEN, PI3K/Akt pathway and cyclin D1 *in* *vitro*. These findings increase understanding of the molecular mechanisms of prostate carcinogenesis and progression, even provide valuable insights into the diagnosis, prognosis, and rational design of novel therapeutics for prostate cancer.

## Introduction

Prostate cancer is one of the most common malignant tumors in men, with the morbidity and mortality significantly increasing in China in recent years, nevertheless the incidence of prostate cancer remains much lower compared to western countries such as Europe and the United States [1-3]. The causes of prostate cancer are complex, with multiple genes involved in its pathogenesis [4]. One of these genes is the tumor-suppressor gene, phosphatase and tensin homologue (PTEN), which is commonly lost or down-regulated in prostate cancers. PTEN locates on chromosome 10q23 and has been shown to play vital roles in the initiation, development and progression of prostate malignancies [4,5]. PTEN is also a member of the protein tyrosine phosphatase (PTP) gene family and influences multiple biological processes such as cell growth, apoptosis, adhesion, migration, and invasion [6]. Consequently, it is important to further elucidate the function and molecular mechanisms of PTEN in tumorigenesis and progression for ultimate application in cancer diagnosis and therapy.

Increasing evidences have demonstrated that microRNAs (miRNAs) play vital roles in the initiation, progression and metastasis of various human cancers by regulation of proto-oncogenes or tumor-suppressor genes [7,8]. Moreover, miRNAs have shown specific expression profilings in particular tumors, which enable microRNAs as biomarkers for diagnosis and prognosis of cancers, and as therapeutic targets in molecular therapy of cancers [8-12]. Increasing numbers of recent studies have indicated that miRNAs are involved in the tumorigenesis and progression of prostate cancer [13]. For instance, Shi et al. found that miR-125b, which aberrantly expressed in prostate cancer cells and tissues, promoted the growth of prostate cancer xenograft through down-regulation of three key pro-apoptotic genes, p53, Puma and Bak1 [14].

In recent years, studies have also increased to investigate the roles of miRNAs in PTEN regulation [6,15]. Meng et al. demonstrated that altered expression of miR-21 promoted cell growth, migration and invasion of liver cancer by regulating PTEN expression [16]. Other studies have also confirmed that miR-21 can promote the growth and metastasis of non-small cell lung cancer [17], gastric cancer [18], and ovarian cancer [19] by inhibition of PTEN expression and regulation of its downstream signals. Furthermore, Iliopoulos et al. showed that miR-21, together with miR-181b-1, underlay the epigenetic switch linking inflammation to cancer by inhibiting PTEN tumor-suppressor function and increasing NF-κB activity [20].

In addition, recent studies have also shown that miR-26a [21], miR-221, miR-222 [22,23], miR-214 [24,25], miR-22 [26], and miR-106b~25 cluster [27] are either directly or indirectly involved in the regulation of PTEN expression, and interfere with tumor growth, progression and radioresistance. Basic mechanisms of miRNA regulation of PTEN expression are still continuously being investigated and new miRNAs that participate in PTEN regulation are still being reported. In this study, we report that four miRNAs (including miR-19b, miR-23b, miR-26a and miR-92a) promote prostate cell proliferation by targeting PTEN, PI3K/Akt pathway and cyclin D1. Our studies provide a novel viewpoint to understand the molecular mechanisms of prostate carcinogenesis and progression, and further contribute to the diagnosis, prognosis and rational design of therapy for prostate cancer.

## Materials and Methods

### Ethics statement

The study was performed in accordance with the Declaration of Helsinki. Written informed consent was obtained from all participants involved in this study. This study was approved by the Institutional Review Board of the School of Life Sciences, Fudan University and Shanghai Jiao tong University School of Medicine, and Hospital Authority of Shanghai First People’s Hospital and Renji Hospital, Shanghai. All the cell lines used in this study were purchased from a commercial source or gifted from other researchers with published references for the origin.

### Plasmids construction

An intact artificial target site capable of simultaneously binding to miR-19b, miR-23b, miR-26a and miR-92a was synthesized by Shanghai Office of Life Technologies Co. (Shanghai, China). This artificial target site (5’-GTTAGATCTGCTAGC ACTGGTCAGTTTTGCATGGATTTGCACATGTG GCAACAGGAGTACAACTAGGTAATCCCTGGCAATGTGATGATATTATTAAGTGCAGCAGCAAGCCTATCCTGGATTACTTAAGGCATCACTTTTTTAATGTTTAACAGGCCGGGACAAGTGCAATATGTTCGATATCAGATCTCAC-3’) ended with BglII, NheI and EcoRV sites for subcloning. The reporter plasmid phEW-luc/PC (positive control) was generated by inserting the BglII-digested fragment above into the phEFW-luc plasmid [28]. The full-length 3’ UTR of PTEN (3302 bp) was amplified by PCR using the following primers: 5’-GTTGCTAGCCTGATCCAGAGAATGAACC T-3’ and 5’-CTCGATATCCCCACACAATGACAAGAATG-3’. The 3’ UTR was double-digested with NheI/EcoRV and inserted into the plasmid phEW-luc/PC to replace the intact artificial target site, and the resulting reporter plasmid was designated as phEW-luc/PU. Similarly, the reporter plasmids phEW-luc/PUA, phEW-luc/PUB, phEW-luc/PUC, and phEW-luc/PUD were constructed by replacing the intact artificial target site with relevant truncated PTEN 3’ UTR fragment A (PUA, 518 bp), fragment B (PUB, 597 bp), fragment C (PUC, 494 bp), or fragment D (PUD, 544 bp) respectively. Accordingly, the mutant counterpart, of which the corresponding miRNA-binding seed region (6 bp) was replaced with a random hexanucleotide (GTCAGT), was synthesized by Shanghai Office of Life Technologies Co. (Shanghai, China). The reporter plasmids containing the mutant counterpart (mPU, mPUA, mPUB, mPUC, or mPUD) were also constructed in similar ways described above. These truncated PTEN 3’ UTR fragments and their mutant counterparts were amplified by PCR using the relevant primers (Table S1) and were further confirmed by PCR sequencing (Sangon, Shanghai, China).

In order to express miR-19b, miR-23b, miR-26a, and miR-92a in prostate cancer cell lines, both expression plasmids pEGP-miR-null and pEGP-miR-NC (Cell BioLabs) were used as vector control and non-effective control (containing a 304 bp mmu-let7a-1 precursor sequence) respectively. The relevant pri-miRNA fragment was first amplified by PCR using the relevant primers (Table S2), and subsequently double-digested with BamHI/XbaI and inserted into the plasmid pEGP-miR-NC in order to replace the intrinsic mmu-let7a-1 precursor fragment. These miRNA expression plasmids were named pEGP-miR-19b, pEGP-miR-23b, pEGP-miR-26a and pEGP-miR-92a respectively. All of the plasmids were confirmed by sequencing (Sangon, Shanghai, China) and purified with the Endo-free Plasmid Midi Kit (QIAGEN) for transfections.

### Cell culture and transfection

Human prostate normal tissues cell line PNT1B [29,30], human prostate cancer cell line LNCaP [31], and human benign prostatic hyperplasia (BPH) epithelial cell line BPH-1 [32] were gifts from Dr. Jia WW (University of British Columbia, Canada) [33]. Human prostate cancer cell lines DU145, PC-3 and 22RV1 [34] were purchased from the Cell Bank of Type Culture Collection of Chinese Academy of Sciences (Shanghai Institute of Cell Biology, Chinese Academy of Sciences, Shanghai, China). PNT1B, BPH-1 and LNCaP were cultured at 37°C in 5% CO_2_ using Dulbecco’s Modified Eagle Medium (DMEM) supplemented with 10% fetal bovine serum (FBS) (GIBCO). On the other hand, DU145 and PC-3 cell lines were cultured in F12 medium, while 22RV1 cell line was cultured in RP1640 medium, both supplemented with 10% FBS. All the cells were seeded on 6-well plates and transfected 24 hrs later using Lipofectamine 2000 (Invitrogen) with 4~5 μg DNA plasmids or a final concentration of 100 nM RNA.

### Luciferase reporter assay

All cells were seeded on 24-well plates and transfected 24 hr later using Lipofectamine 2000. Anti-miR miRNA inhibitor (100 nM, final conc.) (Applied Biosystems) for miR-19b (anti-miR-19b, Cat. No. AM10629), miR-23b (anti-miR-23b, Cat. No. AM10711), miR-26a (anti-miR-26a, Cat. No. AM10249), miR-92a (anti-miR-92a, Cat. No. AM10916) or negative control (Applied Biosystems, Cat. No. AM17010) was co-transfected with 100 ng of luciferase reporter plasmids described above and 2 ng of internal control plasmid pRL-CMV (Promega). Twenty-four hours after transfection, cells were lysed and luciferase activities analyzed using the Dual-Luciferase Assay Reporter System (Promega) according to the manufacturer’s instructions. Reporter luciferase activity was normalized to the internal control *Renilla* luciferase activity in all samples.

### Cell proliferation analysis

After transfection with miRNA expression plasmids, cells were seeded in 24-well plate at 5×10^3^ cells/well and the cell numbers were counted after seeded once a day for one week. The cell density was photographed 4 days after seeding.

### Quantitative reverse transcription polymerase chain reaction (qRT-PCR)

Total RNA was extracted using TRIZOL Reagent (Invitrogen) for reverse transcription. Then cDNAs were synthesized and amplified using relevant cDNA-specific primers (Table S3), SYBR-Green mixture (TOYOBO) and ABI Prism 7000 Sequence Detection System (Applied Biosystems). The relative expression level of target genes was defined as fold changes by 2^-ΔΔCt^ as previously described [28].

For miRNA expression assay, reverse transcription and qRT-PCR were carried out using TaqMan miRNA assay kits (Applied Biosystems) with hsa-miR-19b (Applied Biosystems, Cat. No. 4373098), hsa-miR-23b (Applied Biosystems, Cat. No. 4373073), hsa-miR-26a (Applied Biosystems, Cat. No. 4395166), hsa-miR-92a (Applied Biosystems, Cat. No. 4395169) or U44 (internal control) (Applied Biosystems, Cat. No. 4373384) -specific reverse transcription primers, according to the manufacturer’s instructions. The relative expression level of target genes was defined as fold changes by 2^-ΔΔCt^ as previously described [28].

### Western blot

Cells were harvested and lysed, then subjected to SDS-PAGE electrophoresis and transferred to a polyvinylidene difluoride membrane (Amersham Biosciences) as previously described [28]. The membrane was hybridized with relevant antibodies for visualization using Immobilon^TM^ Western HRP substrate kit (Millipore). The relative quantification was measured by densitometry (Bio-Rad). These antibodies were purchased either from (i) Santa Cruz Biotechnology: antibodies to p110α (Cat. No. sc-7174) and p110δ (Cat. No. sc-7176); or (ii) Cell Signaling Technology: antibodies to p85 (Cat. No. 4292), PTEN (Cat. No. 9188), phospho-PTEN (Cat. No. 9554), AKT (Cat. No. 9272), phospho-AKT (Cat. No. 4060), cyclin D1 (Cat. No. 2978), GAPDH (Cat. No. 2118) and HRP-linked secondary antibodies (Cat. No. 7074).

### Statistical analysis

For comparison of differences between groups, analysis of variance (ANOVA) and unpaired student’s *t* tests were used. Statistical significance was determined by the logrank test. Values of p<0.01 were considered to be statistically significant. Error bars represent the standard error (SE) unless otherwise indicated.

## Results

### Identification of miRNA targets in the PTEN 3’ UTR

**Figure 1:**
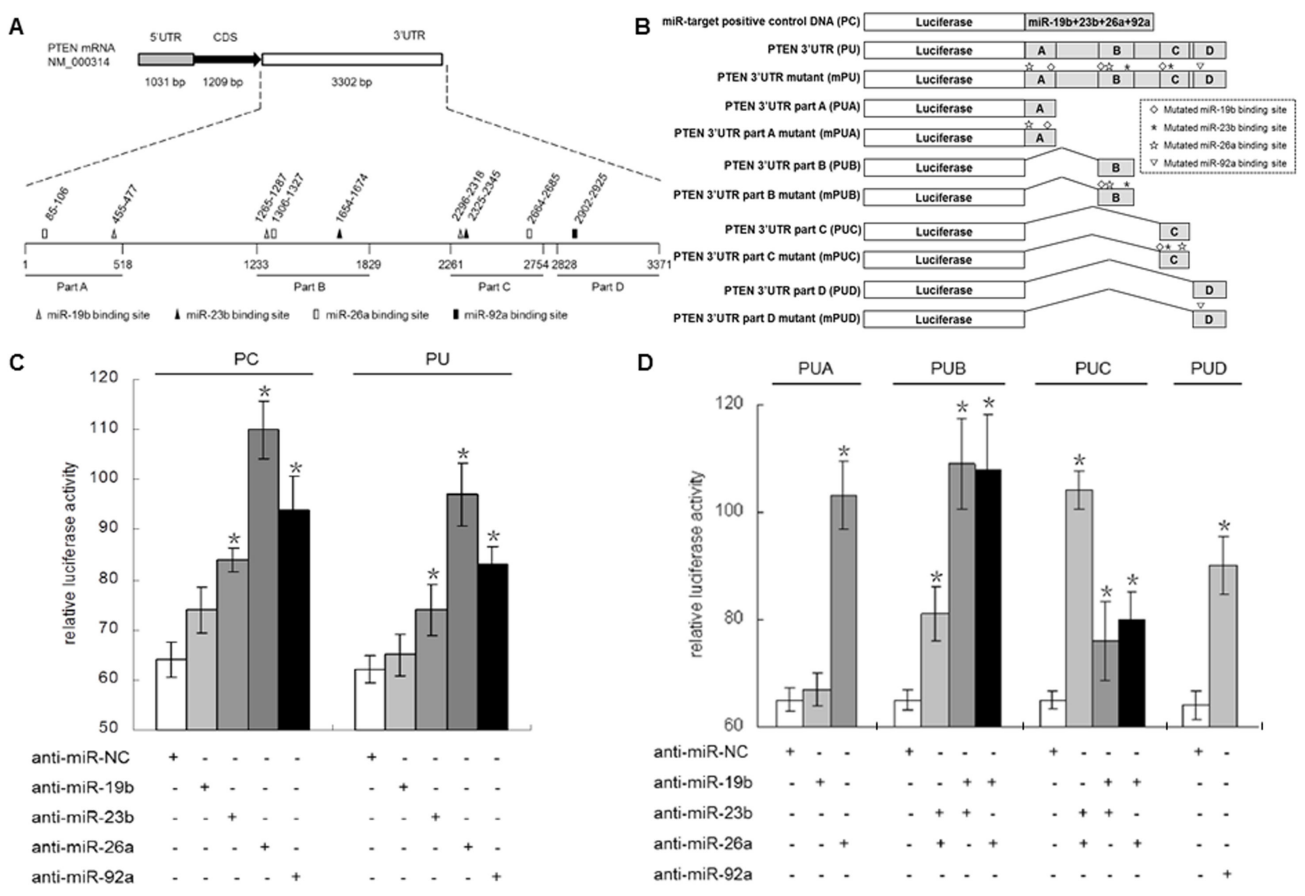
Identification of miRNA targets in the PTEN 3’ UTR. A. Diagram of the PTEN mRNA. Predicted binding sites of four miRNAs (miR-19b, miR-23b, miR-26a and miR-92a) in the 3’ UTR region of PTEN. B. Schematic map of luciferase reporter vectors for measuring the efficacy of the miRNA regulation. C. PNT1B cells were co-transfected the luciferase reporter vectors, which harbored either miR-target positive control (PC) or full-length PTEN 3’ UTR (PU), with relevant anti-miRNA inhibitors or negative control (NC) for the luciferase reporter assay. D. Luciferase reporter vectors containing relevant truncated fragments (PUA, PUB, PUC or PUD) of PTEN 3’ UTR were co-transfected with specific anti-miRNA inhibitors or negative control for the luciferase reporter assay. *indicates a significant difference from the control (p<0.01).

Post-transcriptional regulation via interaction between miRNAs and their target site(s) in 3’ UTRs results in translational repression or mRNA cleavage [35], so putative miRNA target sites within the PTEN 3’ UTR were first determined. Using the online software TargetScanHuman (http://www.targetscan.org/) [36], we predicted multiple putative target sites for two members of miR-23b cluster, miR-26a, miR-23b [37], and two members of the miR-17-92 cluster, miR-19b and miR-92a [38]. These putative target sites were located within four distinct regions of the PTEN 3’ UTR (part A, B, C and D in Figure 1A and Figure S1). For further investigation, we constructed a series of luciferase reporter vectors containing the artificial target sites for the four miRNAs (positive control, PC), full-length PTEN 3’ UTR (PU), its relevant truncated fragment (PUA, PUB, PUC or PUD), and the mutant counterpart (mPU, mPUA,mPUB, mPUC or mPUD), respectively (Figure 1B).

Anti-miR microRNA inhibitor for each miRNA or anti-miR control was co-transfected with the above-mentioned reporter vectors into the human prostate epithelial cell line PNT1B to assess the relative luciferase activity (Figure 1C, 1D, and Figure S2). We found that neutralizing miR-23b, miR-26a or miR-92a with relevant anti-miR inhibitors significantly increased the relative luciferase activity to 1.31±0.04 ~ 1.72±0.09 fold (for PC, Figure 1C, left) or 1.19±0.08 ~ 1.56±0.1 fold (for PU, Figure 1C, right). However, we observed no significant increase of relative luciferase activity when miR-19b was neutralized. This result indicated that compared to miR-19b, the other 3 miRNAs may have stronger interactions with the PTEN 3’ UTR. Meanwhile, we co-transfected the luciferase reporter vectors, which harbored either miR-target positive control (PC), full-length PTEN 3’ UTR (PU), or its mutant counterpart (mPU) respectively, with relevant anti-miRNA inhibitors, and found that the highest relative luciferase activity was achieved to the mutant counterpart (mPU) (Figure S2A).

Next, we cloned 4 truncated fragments (PUA, PUB, PUC or PUD) from different parts of the PTEN 3’ UTR (Figure 1A), each of which contains different miRNA target sites. In part A (PUA), which sequentially contains a miR-26a and a miR-19b target site, we found that neutralizing miR-26a, but not miR-19b, increased the relative luciferase activity to 1.58±0.05 fold (p<0.01, Figure 1D Column I). This suggested that miR-26a had the main effect on interaction with the 3’ UTR in part A. In part B (PUB), which sequentially contains a miR-19b, a miR-26a and a miR-23b target site, we simultaneously neutralized two of the three miRNAs for investigating the regulatory function of the remainder. Our results showed that the relative luciferase activity increased (1.25±0.08 fold, p<0.01) when simultaneously neutralizing miR-23b and miR-26a. This is a significant change but still less than the effect of neutralizing the other two miRNA combinations, namely anti-miR19b-23b (1.68±0.13 fold, p<0.01) and anti-miR-19b-26a (1.66±0.16 fold, p<0.01) (Figure 1D, Column II), which implied that miR-19b had a central role in PUB. In part C (PUC), which sequentially contains a miR-19b, a miR-23b and a miR-26a target site, either miR-23b or miR-26a could repress relative luciferase activity (for anti-miR-19b-23b, 1.17±0.11 fold; for anti-19b-26a, 1.23±0.08 fold; but for anti-23b-26a, 1.61±0.06 fold; p<0.01, Figure 1D Column III), which indicated that both miR-23b and miR-26a had similar levels of interaction with the 3’ UTR. Part D (PUD) contains only a miR-92a target site. The relative luciferase activity increased 1.41±0.09 fold by treatment with an anti-miR-92a inhibitor (p<0.01, Figure 1D Column IV). Accordingly, we synthesized 4 mutant counterparts (mPUA, mPUB, mPUC or mPUD) without the miRNA-target seed region correspondence to the 4 truncated fragments (PUA, PUB, PUC or PUD) (Figure 1B), constructed the corresponding report plasmids, and did the similar transfection experiment and luciferase activity assay as above. We found the mutant counterpart always showed the highest relative luciferase activity, whether the corresponding anti-miR existed or not (Figure S2B, S2C, S2D and S2E). These findings indicated that the four miRNAs (miR-19b, miR-23b, miR-26a and miR-92a) could interact with PTEN 3’ UTR in variant regions for post-transcriptional co-regulation.

### The four miRNAs regulate PTEN expression post-transcriptionally in both prostate cancer and normal prostate epithelial cell lines

**Figure 2:**
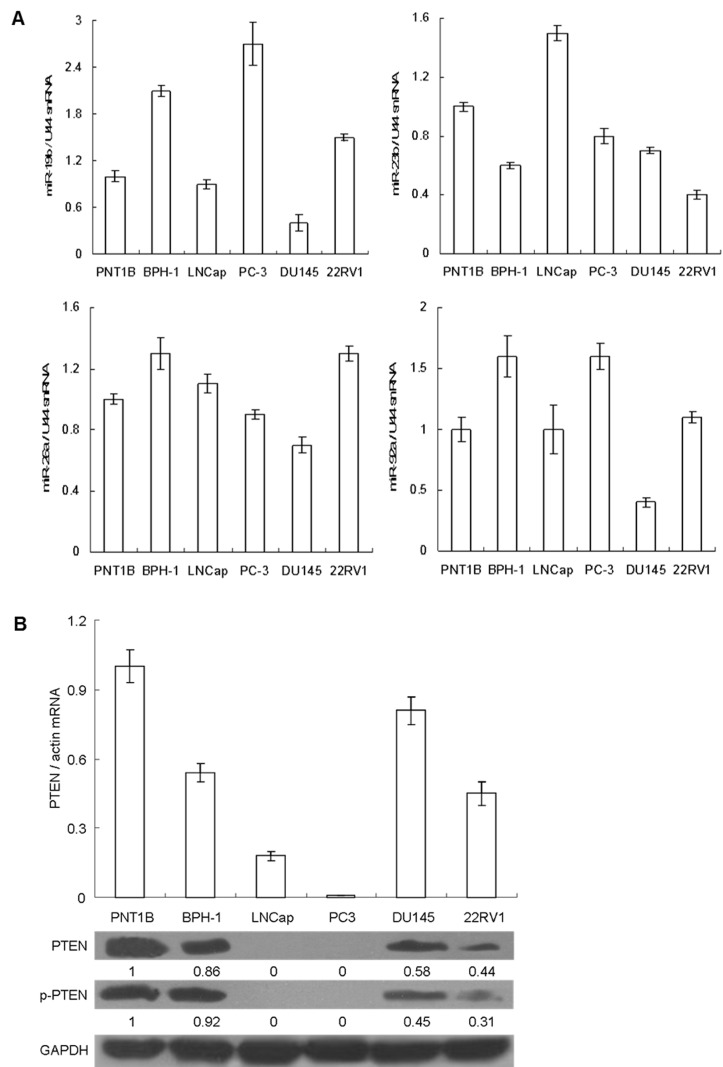
Identification of the endogenous expression of four miRNAs and PTEN in multiple prostate cell lines. A. The endogenous expression of four miRNAs in prostate cancer, BPH and normal prostate epithelial cell line was measured by qRT-PCR. B. The endogenous expression of PTEN in prostate cancer, BPH and normal prostate epithelial cell line was measured by qRT-PCR and western blot. The relative quantification of PTEN protein was measured by densitometry.

We first investigated the endogenous expression of the four miRNAs in PNT1B, a normal prostate epithelial cell line, as well as in several prostate cancer cell lines such as BPH-1, LNCaP, PC-3, DU145, and 22RV1 (Figure 2A). We found that miR-19b expression (Figure 2A, upper left) was up-regulated in BPH-1 (2.07±0.07 fold), PC-3 (2.73±0.28 fold) and 22RV1 (1.46±0.05 fold), but down-regulated in DU145 (0.41±0.11 fold), relative to PNT1B levels. The expression of miR-23b (Figure 2A, upper right) was down-regulated in nearly all prostate cancer cell lines including BPH-1 (0.62±0.02 fold), PC-3 (0.78±0.05 fold), DU145 (0.66±0.02), 22RV1 (0.37±0.03 fold), but up-regulated in LNCaP (1.52±0.05 fold). On the other hand, miR-26a expression (Figure 2A, lower left) was the highest in 22RV1 (1.32±0.05 fold) and the lowest in DU145 (0.73±0.05 fold), while miR-92a (Figure 2A, lower right) was up-regulated in both BPH-1 (1.62±0.17 fold) and PC-3 (1.57±0.11 fold), but down-regulated in DU145 (0.41±0.04 fold). Aside from measuring endogenous miRNA levels, we also investigated endogenous PTEN expression in all relevant cell lines. PTEN expression was undetectable in LNCaP and PC-3 [39], while being significantly repressed in BPH-1, DU145 and 22RV1 cell lines (Figure 2B). Among the latter three prostate cancer cell lines, PTEN expression was the highest in DU145, which corresponded with the down-regulation of endogenous four miRNAs in these cells (Figure 2A). Based on these findings, we wondered whether subtle relationships might exist between expression profiles of four miRNAs and PTEN in DU145 cells.

**Figure 3:**
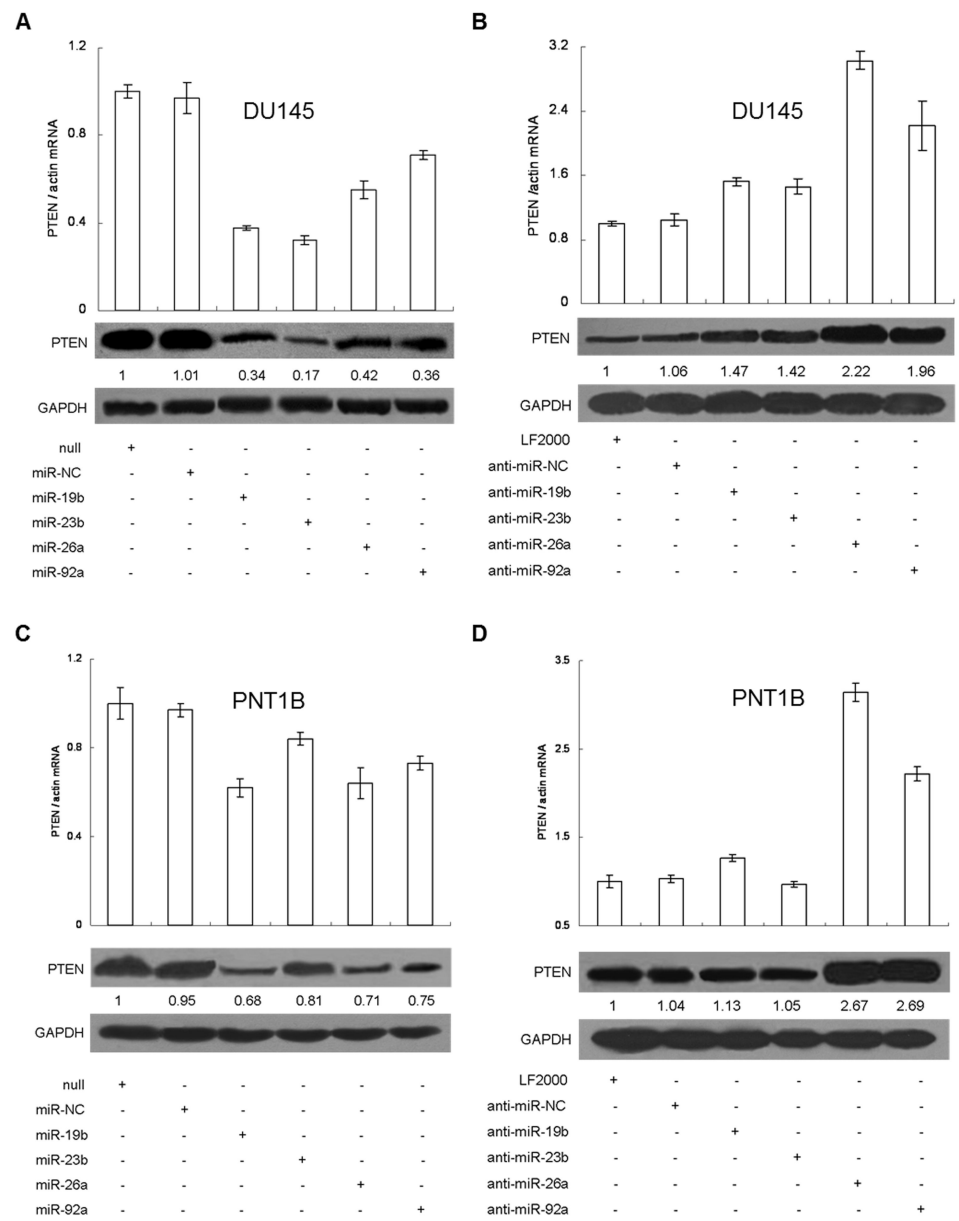
The four miRNAs co-regulate PTEN expression. Overexpression of miR-19b, miR-23b, miR-26a or miR-92a repressed PTEN expression in DU145 (A) and PNT1B (C), while neutralization of each miRNA alone up-regulated PTEN expression in DU145 (B) and PNT1B (D). The expression of PTEN was measured by qRT-PCR and western blot. The relative quantification of PTEN protein was measured by densitometry.

We proceeded to transfect the miRNA expression vectors into DU145 and found that the total expression of relevant miRNA increased by more than 2-fold (Figure S3A). We further found that overexpression of any one of the four miRNAs could effectively repress (0.32±0.02 ~ 0.71±0.02 fold) PTEN expression (Figure 3A). On the other hand, cells were also treated with anti-miRs in order to neutralize the four miRNAs (Figure S3B), resulting in the up-regulation of PTEN expression (1.46±0.1 ~ 3.03±0.11 fold). Among the four miRNAs targeted, inhibition of miR-26a or miR-92a resulted in a more significant and dramatic up-regulation of PTEN expression (Figure 3B), despite their modest repressive abilities (0.5 and 0.7 fold when overexpression of miR-26a and miR-92a, respectively) (Figure 3A). We repeated miRNA overexpression (Figure S4A) and neutralization (Figure S4B) experiments in normal prostate PNT1B cells and found similar responses for PTEN expression (Figure 3C and 3D). Furthermore, we attempted to neutralize two or three of the four miRNAs simultaneously with the relevant anti-miR inhibitors in PNT1B cells (Figure S5A, S5B). PTEN expression increased dramatically upon inhibition of miR-26a and/or miR-92a expression (Figure S5). We also used PTEN-specific siRNAs as a positive control to confirm the specificity of the 4 miRNA-mediated PTEN repressions in both DU145 and PNT1B cells (Figure S6). These findings demonstrate that four miRNAs can regulate PTEN expression post-transcriptionally in both normal prostate and prostate cancer epithelial cell lines, with miR-26a and miR-92a potentially playing more important roles among the four miRNAs.

### The four miRNAs participate in the regulation of PI3K/Akt pathway

**Figure 4:**
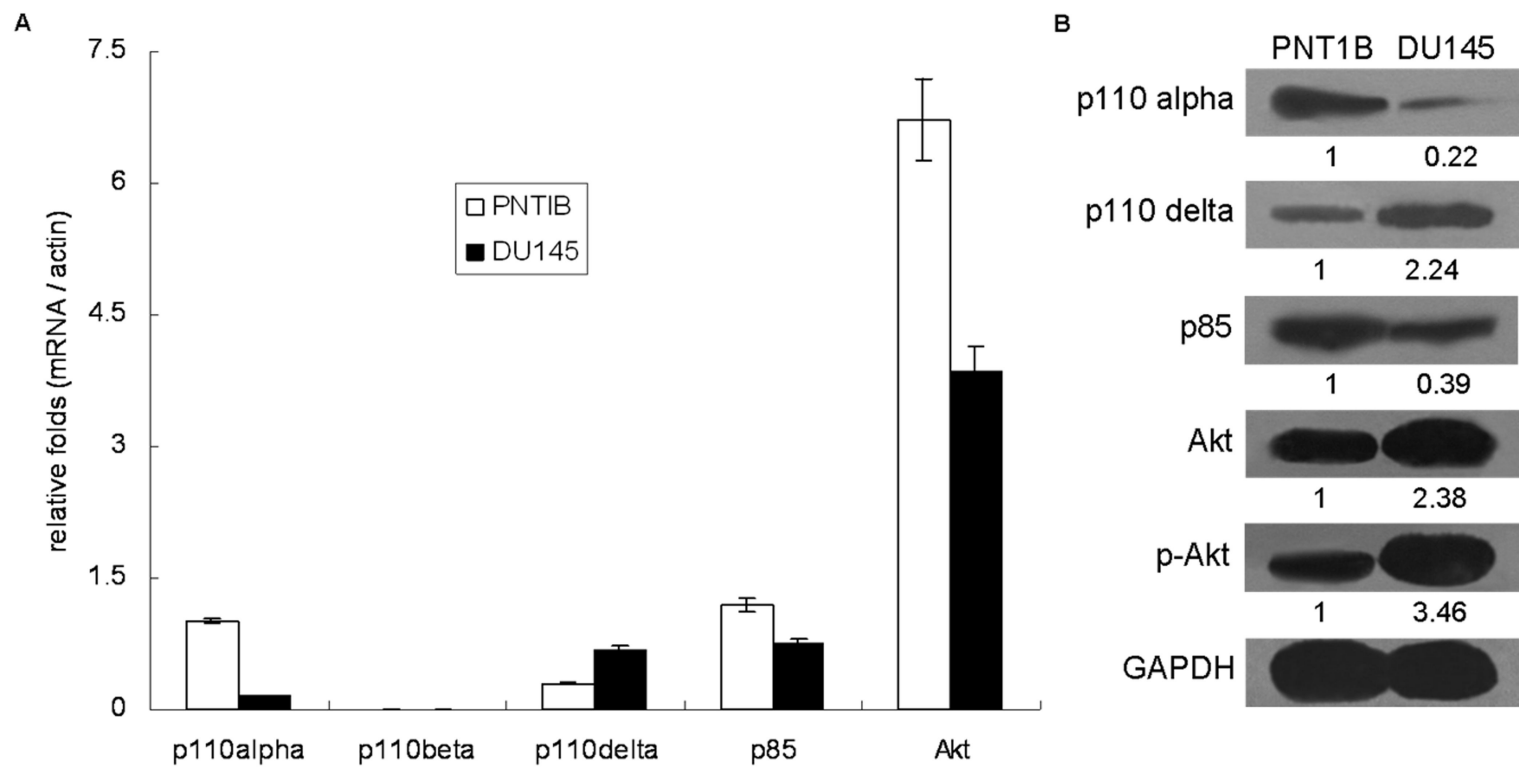
Endogenous expression of p110α, p100β, p110δ, p85 and Akt in DU145 and PNT1B cell lines. A. The mRNA expressional level of the 5 genes was measured by qRT-PCR. B. the protein expressional level was measured by western blot. The relative quantification of PTEN protein was measured by densitometry.

We investigated the interaction between the four miRNAs and the PI3K/Akt pathway, which is a key cell proliferation pathway regulated by PTEN [40]. Specifically, we focused on i) the three different Class IA catalytic subunits of PI3-kinase, namely p110α, p110β and p110δ, ii) the regulatory subunit of PI3-kinase, p85, and iii) the downstream responsive component, Akt [41]. First, we compared the endogenous expression of these genes in DU145 and PNT1B (Figure 4). Except for the p110β, all the other genes were expressed in different levels in both cell lines. In PNT1B cells, the main catalytic subunit was p110α (5- to 6-fold more than in DU145), while in DU145 cells, it was p110δ (2-fold more than in PNT1B). In addition, both the protein expression and activity of Akt were stronger in DU145, although the Akt mRNA level was higher in PNT1B by unknown reasons (Figure 4). We also found putative target sites of the four miRNAs located in the 3’ UTR of p110α, p110δ, p85 and Akt (Table S4 and Figure S7), which indicated that these miRNAs might further participate in the post-transcriptional regulation of these genes.

**Figure 5:**
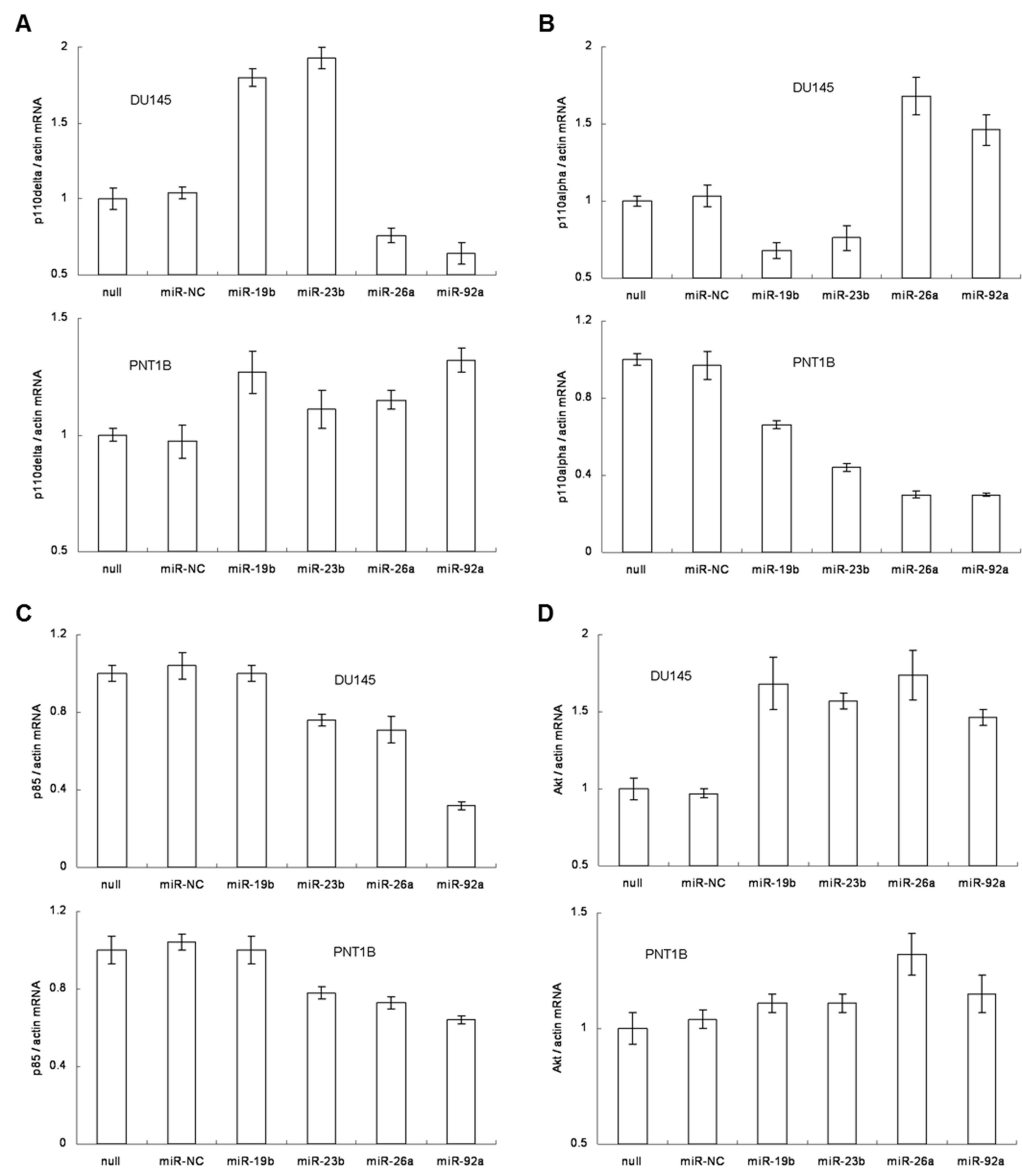
The alteration of mRNA expression level in miRNA overexpressed DU145 and PNT1B cells. The mRNA expression level of p110δ (A), p110α (B), p85 (C) and Akt (D) was measured by qRT-PCR and appeared to be altered when the relevant miRNA was overexpressed in DU145 or PNT1B cells.

We transiently transfected miR-19b, miR-23b, miR-26a or miR-92a expression vectors in DU145 and PNT1B cells, then measured expression levels of p110α, p110δ, p85 and Akt mRNA by qRT-PCR (Figure 5). Given that p110δ expression directly responds to PTEN [42], which is in turn regulated by miRNAs, we first investigated the alteration of p110δ expression after overexpressing the four miRNAs (Figure 5A). In both DU145 and PNT1B cells, overexpression of either miR-19b or miR-23b alone repressed PTEN expression (Figure 3A) and thus relieved its repression of p110δ, thereby increasing p110δ expression by 1.5-fold. Since p110δ can be regulated by several factors including PTEN, miR-26a and miR-92a (Table S4 and Figure S7), its final expression level depends on the total influence of the antagonistic regulators. Hence, p110δ expression was still repressed in DU145, but restored in PNT1B after overexpressing either miR-26a or miR-92a (Figure 5A). For p110α, its expression level was not only complementary with that of p110δ [43], but also regulated by miR-19b (Table S4 and Figure S7). So in DU145 cells, overexpression of either miR-19b or miR-23b resulted in repression of p110α, while overexpression of either miR-26a or miR-92a increased p110α expression. In PNT1B cells, p110α expression was repressed when overexpressing miR-19b, miR-23b, miR-26a or miR-92a (Figure 5B). On the other hand, post-transcriptional regulation of p85 expression was only associated with overexpression of miR-23b, miR-26a or miR-92a (Table S4 and Figure S7). We found that overexpression of these three miRNAs could down-regulated p85 expression in the both cell lines (Figure 5C). For Akt, its expression level depended on both the total expression of the PI3-kinase catalytic subunits [44] and miR-26a (Table S4 and Figure S7). In DU145 cells, Akt expression was increased by overexpressing any of the four miRNAs. However, only when overexpressing the miR-26a, a significant increase of Akt expression was observed in PNT1B cells (Figure 5D). Finally, it is noteworthy to mention that the protein levels were consistent with the mRNA levels after overexpressing the four miRNAs (Figure S8).

In order to validate the observations above, we further employed PTEN inhibitor and PTEN siRNA#2 to repeat the experiments (Figure S9). The results showed that PTEN inhibitor or siRNA#2 only affected the activity or expression of PTEN but did not regulate p110α, p110δ, p85 or Akt levels directly. We found that either inhibitor (Figures S9A and S9B) or siRNA#2 (Figures S9C and S9D) could indirectly improve the expression of p110α, p110δ, p85 and Akt on a higher level than overexpressing relevant miRNAs (compare Figure 5 to Figure S9). These results indicate that four miRNAs participated in the regulation of both PTEN and the key components of the PI3K/Akt pathway in normal and cancerous prostate epithelial cell lines. Our results also suggested that among the investigated miRNAs, miR-26a may have a more significant role in up-regulating the expression of PI3K/Akt pathway.

**Figure 6:**
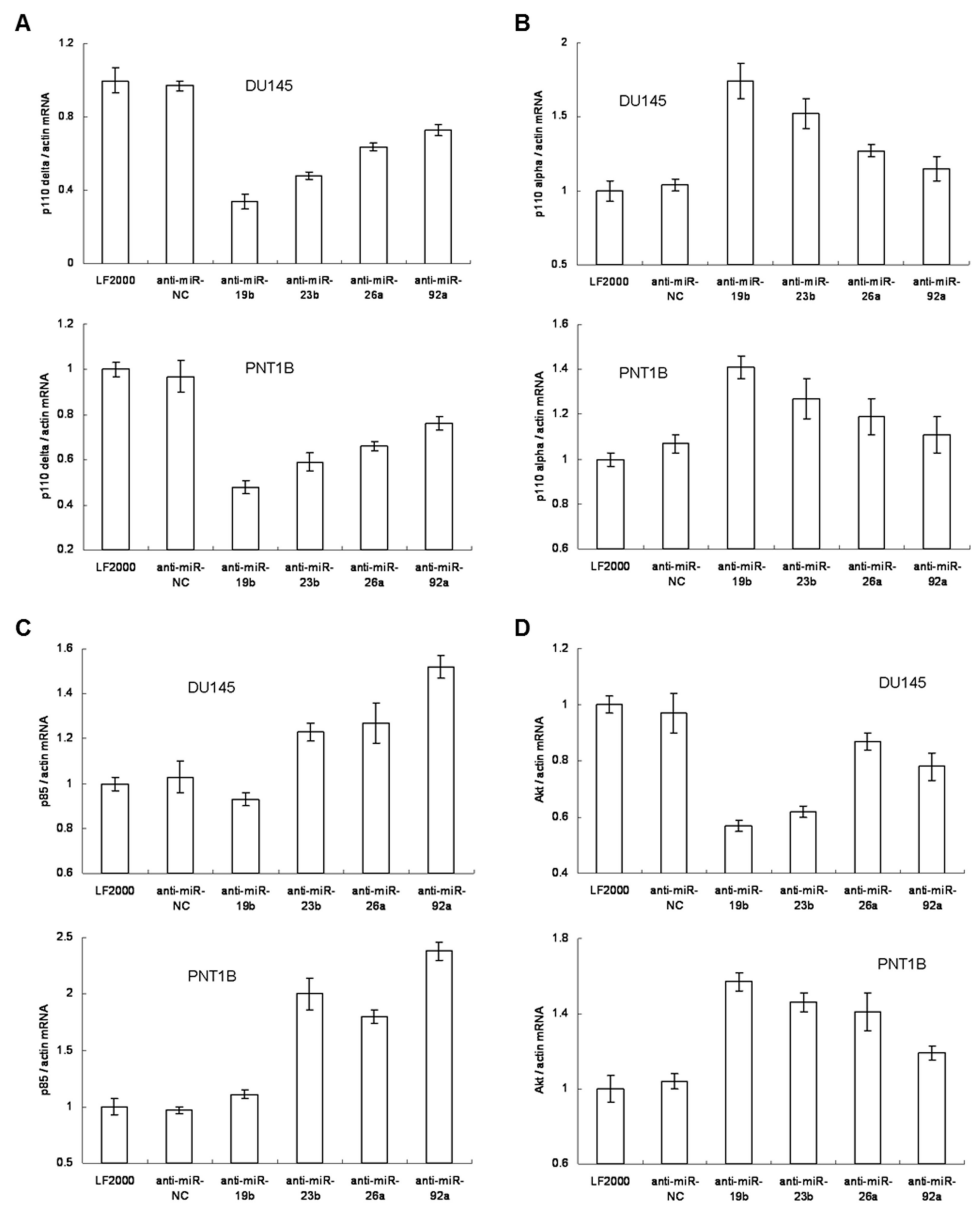
The alteration of mRNA expression level in miRNA inhibited DU145 and PNT1B cells. The mRNA expression level of p110δ (A), p110α (B), p85 (C) and Akt (D) was measured by qRT-PCR and appeared to be altered when the relevant miRNA was inhibited in DU145 or PNT1B cells.

In addition to overexpression of the four miRNAs, we also neutralized these miRNAs using anti-miR inhibitors and subsequently analyzed the effects on the PI3K/Akt pathway components (Figure 6). For p110δ, all four anti-miR inhibitors repressed p110δ expression in both DU145 and PNT1B. Intriguingly, anti-miR-26a and anti-miR-92a could also neutralize the repressive effects of miR-26a and miR-92a for p110δ expression. This resulted in the final p110δ level being higher in cells treated with anti-miR-26a or anti-miR-92a compared to those with anti-miR-19b or anti-miR-23b (Figure 6A). For p110α, mRNA expression was increased after imposing any of the four anti-miR inhibitors, which showed an opposite trend compared to the observation in p110δ (Figure 6B). For p85, neutralizing the four miRNAs increased mRNA expression, except for miR-19b (Figure 6C). This is an expected result that is complementary to and in agreement with our observations in the miRNA overexpression experiments (Figure 5C), and demonstrates that p85 expression is regulated by miR-23b, miR-26a and miR-92a at the post-transcriptional level and is not associated with PTEN expression (Figure 6C). Finally, Akt levels were also repressed by neutralizing relevant miRNAs with anti-miR inhibitors, consistent with the down-regulation of total expression of PI3-kinase catalytic subunits. Among the four miRNAs, anti-miR-26a reduced the repressive function of miR-26a on Akt expression most effectively, resulting in the least down-regulation after DU145 cells were treated with the anti-miR-26a inhibitor (Figure 6D). In contrast, Akt expression increased by treatment with all four anti-miR inhibitors in PNT1B cells by a similar mechanism. Importantly, the protein levels accurately reflected mRNA levels after miRNA neutralization (Figure S10). These results again prove that the four miRNAs co-regulated the expression of both PTEN and the key components of the PI3K/Akt signaling pathway in normal prostate and prostate cancer epithelial cell lines. These findings also reveal the complexity of miRNA regulation of PTEN expression and its relevant downstream signals.

### The four miRNAs co-regulate cyclin D1 expression in both prostate cancer and normal prostate epithelial cell lines

**Figure 7:**
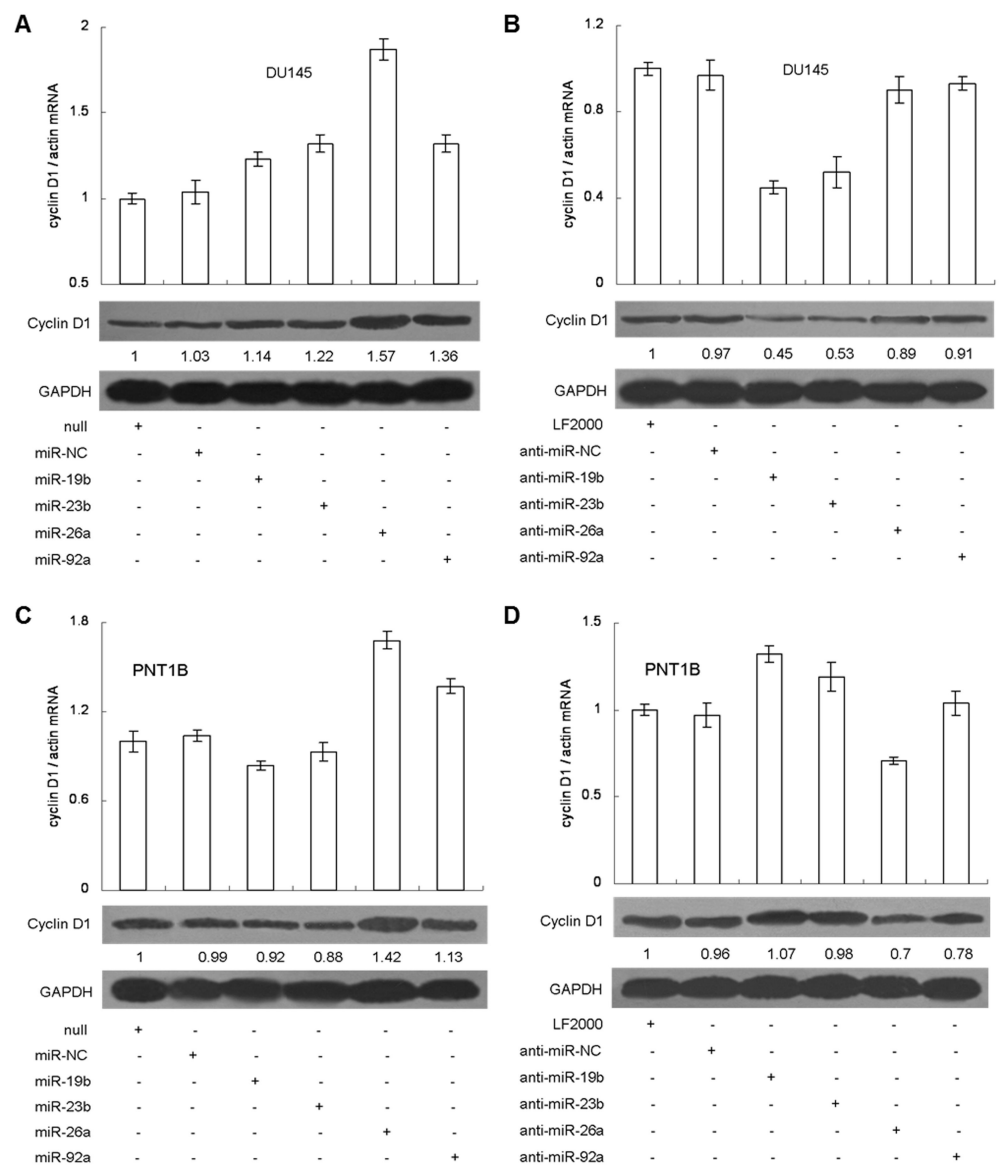
The four miRNAs co-regulate cyclin D1 expression. Overexpression of miR-19b, miR-23b, miR-26a or miR-92a up-regulated cyclin D1 expression in DU145 (A) and PNT1B (C), while neutralization of each miRNA alone repressed the cyclin D1 expression in DU145 (B) and PNT1B (D). The expression of cyclin D1 was measured by qRT-PCR and western blot. The relative quantification of PTEN protein was measured by densitometry.

Cyclin D1 is an important downstream component regulated by PI3K/Akt signaling [45] and is usually overexpressed in prostate cancer [46]. Hence, we wondered whether the four miRNAs could regulate cyclin D1 expression. Using bioinformatics analysis, we found several miRNA target sites, including three miR-19b, one miR-23b and one miR-92a located in the cyclin D1 3’ UTR (Figure S11A). We also identified that the endogenous expression of cyclin D1 was dramatically higher in DU145 than in PNT1B cells (Figure S11B). In DU145, four miRNAs increased cyclin D1 expression after overexpression, of which miR-26a showed the best effect because of the absence of relevant target site in cyclin D1 3’ UTR (Figure 7A). After neutralization by anti-miR inhibitors, cyclin D1 expression was repressed significantly by anti-miR-19b and anti-miR-23b, but only slightly by anti-miR-26a and anti-miR-92a (Figure 7B). In PNT1B, a similar increase of the cyclin D1 expression was observed when miR-26a or miR-92a was overexpressed, but only a slight repression when miR-19b or miR-23b was used (Figure 7C). When anti-miR-19b or anti-miR-23b inhibitors were used, they showed no change in cyclin D1 protein expression relative to control, yet showed an increase in mRNA expression. Meanwhile, anti-miR-26a or anti-miR-92a inhibitor repressed cyclin D1 protein expression, although its mRNA expression was similar to the control (Figure 7D). Moreover, we also observed the increase of cyclin D1 expression in both DU145 and PNT1B when PTEN inhibitor or siRNA#2 was imposed (Figure S12). These results indicate that cyclin D1 expression can also be regulated by the four miRNAs as well as in an Akt-dependent manner.

### Overexpression of the four miRNAs stimulate cell proliferation in DU145 and PNT1B

**Figure 8:**
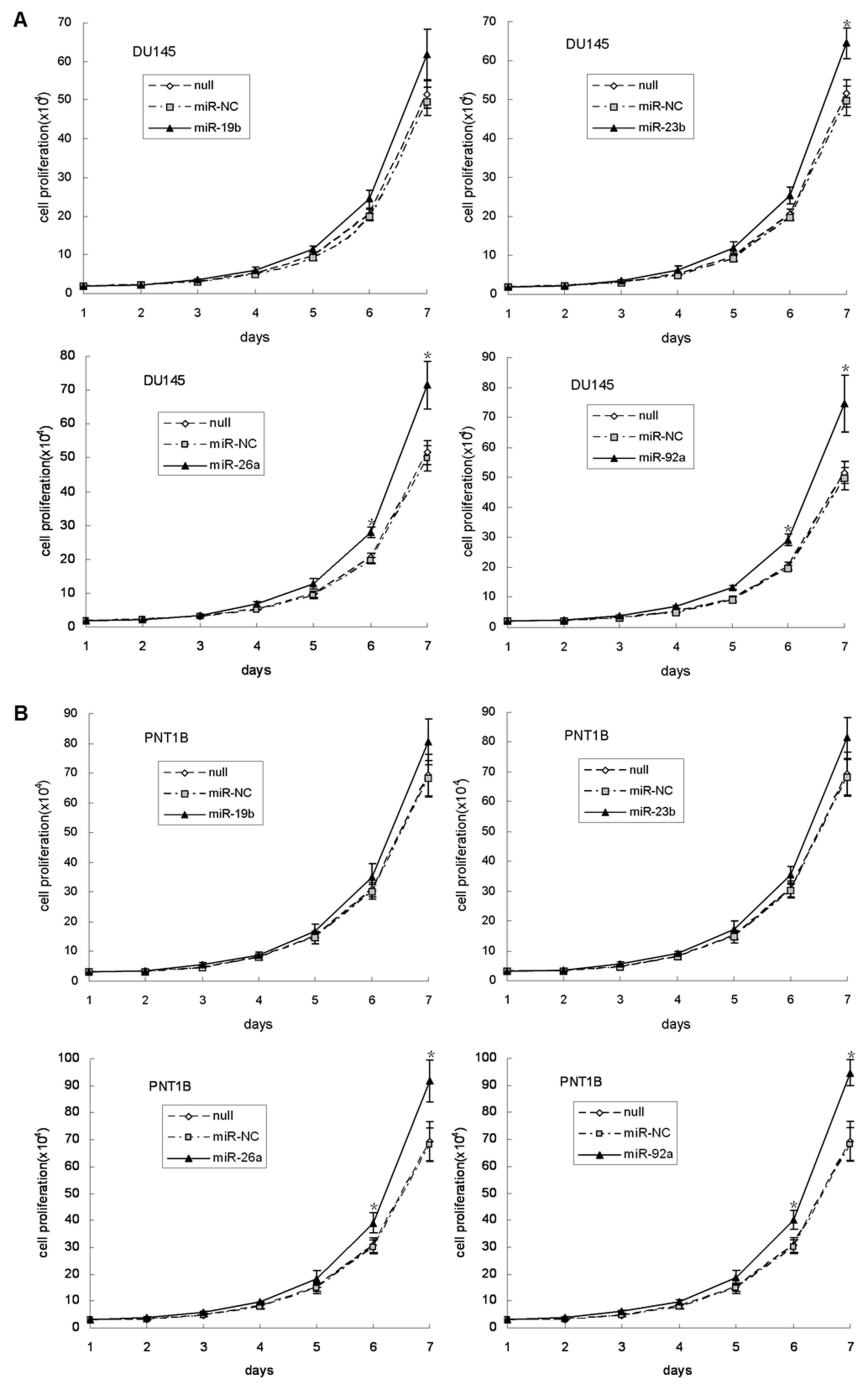
Cell proliferation assay of DU145 and PNT1B cells after overexpression of the four miRNAs. Overexpression of miR-19b, miR-23b, miR-26a, or miR-92a stimulated cell proliferation in DU145 (A) and PNT1B (B). *indicates a significant difference from the control (p<0.01).

The effect of overexpression of the four miRNAs on stimulating cell proliferation in prostate cancer and prostate epithelial cells was investigated *in vitro*. The cell numbers were counted once a day for one week and cell growth observed by micrograph on Day 4 after cell seeding (Figure S13). We found that cell proliferation could be significantly stimulated in DU145 by overexpressing any of the four miRNAs, except for miR-19b (Figure 8A). Similar results were obtained using PTEN inhibitor or siRNA#2 as control (Figure S14). In PNT1B, cell proliferation was likewise observed after overexpressing four miRNAs. Using micrograph (Figure S15) and cell counting, it was demonstrated that any of the four miRNAs could enhance cell growth, with miR-26a and miR-92a having the most significant effect on cell proliferation (Figure 8B). A similar result was also observed after imposing PTEN inhibitor or siRNA#2 (Figure S16). We further observed the cell growth after overexpression of miR-26a, or its combination with miR-19b, miR-23b, or miR-92a in prostate cells, and found that overexpression of the combination of miR-26a and miR-92a would significantly promote prostate cell proliferation, nevertheless overexpression of miR-26a combination with miR-19b, or miR-23b showed moderate cell proliferation (Figure S17). These results indicate that overexpression of the four miRNAs may act as oncogenic miRNAs and could stimulate the cell proliferation in both prostate cancer and epithelial cell.

## Discussion

PTEN is a biomarker for prostate cancer prognosis, in which lower PTEN protein expression associated with more advanced malignancies and poorer prognosis [4,5]. At the cellular level, PTEN, which has bi-specific (lipid and protein) phosphatase activity, can dephosphorylate PIP3 to PIP2 to keep cellular PIP3 levels low, resulting in inhibition of the PI3K/Akt signaling pathway and maintenance of the normal cell cycle. On the other hand, down-regulation of PTEN, such as caused by mutation, deletion, etc., would cause cellular PIP3 levels to increase and subsequently over-activate the PI3K/AKT pathway, which ultimately lead to uncontrolled cell proliferation and tumorigenesis [47-49].

Although PTEN structure, function, expression and regulation mechanisms have been well studied and documented in literature, a lot of questions remain and are worth of further investigation [6]. In addition to well-known genetic alterations of PTEN, epigenetic silencing and transcriptional regulation, recently post-transcriptional regulation of PTEN by non-coding RNAs has attracted much attention. Some studies have explored the interactions between miRNAs and PTEN expression as mentioned above [15-19]. In this study, we sought to identify which miRNAs impose post-transcriptional regulation of PTEN expression and what additional effects they may produce in prostate cancer cells. Our results found that four miRNAs, which include miR-19b, miR-23b, miR-26a and miR-92a, promote cell proliferation of prostate cancer *in vitro* by targeting regulation of PTEN and its downstream signals, PI3K/Akt and cyclin D1.

MiR-19b and miR-92a are members of the miR-17-92 cluster, which plays an important role in the process of tumor initiation and progression [38]. Kurokawa et al. found that miR-19b was up-regulated 3.5-fold in the 5-fluorouracil (5-FU)-resistant colon cancer cells, and potentially regulated the cell cycle regulators, splicing factor proline/glutamate-rich (SFPQ) and v-Mybmyeloblastosis viral oncogene homolog-like 2 (MYBL2) [50]. Olive et al. considered miR-19 to be the key oncogenic component of the miR-17-92 cluster, wherein miR-19 (including miR-19a and miR-19b) partially suppressed PTEN expression, activating the Akt-mTOR (mammalian target of rapamycin) signaling pathway and promoting cell survival [51]. These results from previous studies were in accordance with results from this work, in which we found that miR-19b engaged in the regulation of PTEN expression in prostate cancer cells, but showed a relatively subordinate role in the process.

MiR-92 was previously thought to control angiogenesis, and inhibition of miR-92a was believed to help ischemia recovery in mice [52]. Later studies showed that miR-92a inhibited angiogenesis of endothelial cells mainly by mediating regulation of Kruppel-Like Factor (KLF) 4 and KLF2 expression [53,54]. Tsuchida et al. considered miR-92 to be a key component of the miR-17-92 cluster in colon cancer, which directly regulated the expression of the anti-apoptotic molecule BCL-2 -interacting mediator of cell death (BIM) [55]. Chen et al. also found that overexpression of miR-92a promoted lymph node metastasis via E-cadherin in human esophageal squamous cell carcinoma [56].

MiR-23b and miR-26a are considered to be members of the miR-23b cluster [37]. Salvi et al. found that overexpression of miR-23b suppressed the translation of urokinase-type plasminogen activator (uPA) and c-Met, and even decreased migration and proliferation of hepatocellular carcinoma (HCC) cells [57]. MiR-23b was also found to regulate transforming growth factor-beta (TGF-β) / bone morphogenetic protein (BMP) signaling through targeting Smads (drosophila mothers against decapentaplegic protein) and influenced liver stem cell differentiation [37]. Moreover, miR-23b could regulate retinoblastoma phosphorylation, induce cell cycle arrest at G0/G1 phase, and inhibit vascular endothelial cell proliferation [58]. Zhang et al. demonstrated that miR-23b is down-regulated in human colon cancer and mediates multiple steps of metastasis, including tumor growth, invasion and angiogenesis by regulating a cohort of prometastatic targets, including Frizzled-7 and MAP3K1 (mitogen-activated protein kinase kinase kinase 1) [59].

MiR-26a has been always drawn much attention in cancer research. Huse et al. demonstrated that miR-26a, which was most often associated with monoallelic PTEN loss and directly regulated PTEN expression, facilitated gliomagenesis and was frequently amplified in high-grade gliomas [21]. However, Kota et al. found that HCC cells exhibited reduced expression of miR-26a, which was normally expressed at high levels in diverse tissues. Overexpression of miR-26a induced cell-cycle arrest by direct targeting of cyclins D2 and E2. Furthermore, adeno-associated virus (AAV)-mediated miR-26a restrained cancer cell proliferation, induced tumor-specific apoptosis, and significantly protected against disease progression (without toxicity) in an HCC mouse model [11]. The level of miR-26 (miR-26a and miR-26b) expression was associated with HCC, and HCC with reduced miR-26 expression exhibited a distinct transcriptome pattern by activating the signaling pathways between NF-κB and IL-6 [10]. Furthermore, miR-26a, driven by a hAFP (human alpha fetoprotein) -hTERT (human telomerase reverse transcriptase) dual promoter, was found to specifically decrease the viability of liver cancer cells by regulating estrogen receptor, progesterone receptor and p53 (but not cyclin D2 or cyclin E2) *in vitro* and *in vivo* [60].

**Figure 9:**
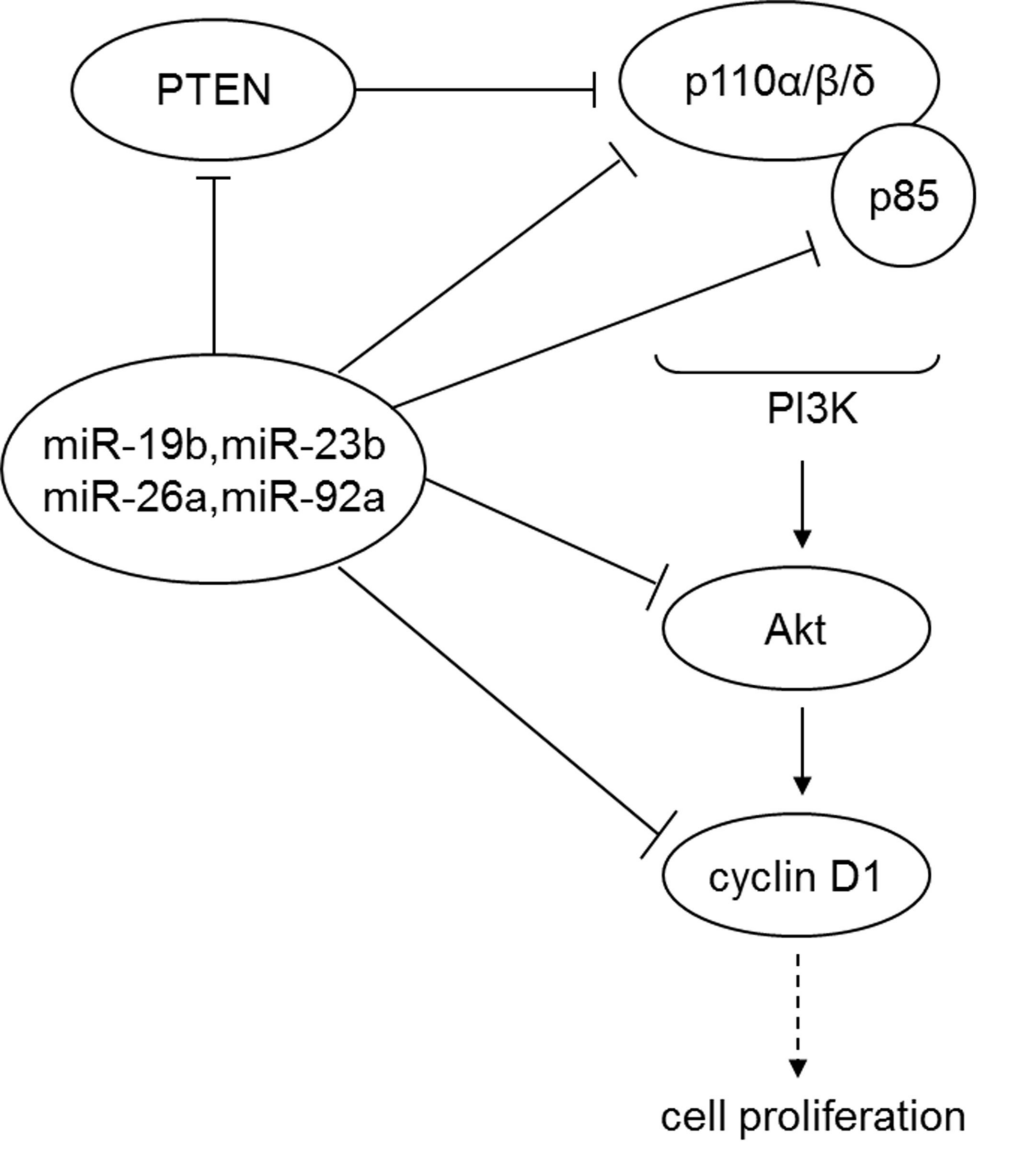
Schematic model of a putative mechanism for prostate cancer cell proliferation via microRNA regulation of PTEN and its downstream signals. Increased amount of miR-19b, miR-23b, miR-26a and miR-92a may impose inhibitory effects on PTEN, PI3K/Akt signaling components, and cyclin D1, respectively. The expression level of PI3-kinase, Akt and cyclin D1 was associated with the expression level of relevant upstream regulators and the regulation of the relevant miRNAs.

Driven by the hypothesis that PTEN-binding miRNAs may regulate this signaling pathway, we report for the first time that the four miRNAs, including miR-19b, miR-92a, miR-23b and miR-26a, promote prostate cancer cell proliferation by regulating the expression of PTEN and its downstream signals, PI3K/Akt and cyclin D1. We also found that the four miRNAs had one or more target sites in the key components of the PI3K/Akt pathway including PIK3CA (p110α, PIK3CD (p110δ), PIK3R1 (p85) and Akt (Figure 5, 6, S6, and Table S4), and cyclin D1 (Figure 8, S10). This suggests that the miRNAs not only co-regulate and suppress PTEN expression in prostate cancer cells, but also coordinately affect the key components of PI3K/Akt and cyclin D1. On the other hand, since PTEN can directly inhibit the PI3K/Akt signaling pathway, there might exsit a complex balance between the neutralizing vs. reinforcing effects of upstream gene regulation, which would require further studies to draw a complete picture about the regulatory network. Based on their more dominant effects on PTEN level, we suggest that miR-26a and miR-92a might act as oncogenes in prostate cancer and play a more important role in co-regulation of the four miRNAs (Figure 9). Intriguingly, the competing endogenous RNA (ceRNA) hypothesis proposed by Salmena et al. in 2011 pointed to some new directions for future exploration of the complex mechanisms of PTEN regulation [61].

In conclusion, we herein proposed for the first time that the four miRNAs, i.e. miR-19b, miR-23b, miR-26a and miR-92a, co-regulate PTEN expression in prostate cancer cells. The four miRNAs regulate PTEN expression post-transcriptionally, and affect the downstream PI3K/Akt pathway via PIK3CA (p110α), PIK3CD (p110δ), PIK3R1 (p85), Akt as well as cyclin D1, thus promote prostate cancer cell proliferation *in vitro*. These findings help to better understand the molecular mechanism of prostate carcinogenesis and progression with regard to miRNA regulation of PTEN expression, and may provide insights for better diagnosis, therapy and prognosis of prostate cancer in the future.

## Supporting Information

Table S1 **Primers for amplifying truncated PTEN 3’ UTR fragments and their mutant counterparts.**
(DOC)

Table S2 **Primers for amplifying pri-miRNA fragments.**
(DOC)

Table S3 **Primers for qRT-PCR.**
(DOC)

Table S4 **Target sites of the four miRNAs in the 3’UTR of the key components of PI3K/Akt pathway.**
(DOC)

Figure S1 **Sequences of RNA-target sites for the four miRNAs in the PTEN 3’ UTR.** Sequences of RNA-target sites for miR-19b (A), miR-23b (B), miR-26a (C) and miR-92a (D) in the PTEN 3’ UTR are shown paired with specific miRNA sequences. The seed region of each miRNA is underlined.
(DOC)

Figure S2 **Validation of miRNA targets in the PTEN 3’ UTR.** (A) PNT1B cells were co-transfected the luciferase reporter vectors, which harbored either miR-target positive control (PC), full-length PTEN 3’ UTR (PU), or its mutant counterpart (mPU) respectively, with relevant anti-miRNA inhibitors for the luciferase reporter assay. (B-E) Luciferase reporter vectors containing relevant truncated fragment PUA (B), PUB (C), PUC (D) or PUD (E) of PTEN 3’ UTR with (MT) or without (WT) mutant(s) in its relevant miRNA binding site(s) were co-transfected with specific anti-miRNA inhibitors respectively for the luciferase reporter assay. *indicates a significant difference from the control (p<0.01).
(DOC)

Figure S3 **TaqMan microRNA assay for the specific miRNA expression in DU145.** (A) The four miRNAs were overexpressed after transient transfection of the specific miRNA expression vectors. (B) The four miRNAs were neutralized after the specific anti-miRNA inhibitors were imposed.
(DOC)

Figure S4 **TaqMan microTNA assay for specific miRNA expression in PNT1B.** (A) The four miRNAs were overexpressed after transient transfection of specific miRNA expression vectors. (B) The four miRNAs were neutralized after the specific anti-miRNA inhibitors were imposed.
(DOC)

Figure S5 **Up-regulation of PTEN expression by neutralizing the specific miRNAs in PNT1B.** The expression of PTEN was up-regulated after two (A) or three (B) of four miRNAs were neutralized simultaneously in PNT1B. The relative quantification of PTEN protein was measured by densitometry.
(DOC)

Figure S6 **Repression of PTEN by PTEN-specific siRNA interference.** PTEN expression was repressed by PTEN-specific siRNAs in DU145 (A) and PNT1B (B), and among them PTEN siRNA#2 was identified as the most effective silencer for the following experiments and to be used as a positive control. The relative quantification of PTEN protein was measured by densitometry.
(DOC)

Figure S7 **Prediction diagram of miRNA targeting site in the PIK3CA (p110α), PIK3CD (p110δ), PIK3R1 (p85) and Akt mRNA 3’ UTR.** PIK3CA 3’ UTR harbors a miR-19b targeting site, while PIK3CD 3’ UTR harbors a miR-26a and a miR-92a targeting site. In the PIK3R1 3’ UTR, multiple miR-23b, miR-26a and miR-92a targeting sites can be found. In Akt 3’ UTR, a miR-26a targeting site is located.
(DOC)

Figure S8 **The protein expression level of p110α, p110δ, p85 and Akt was altered after the relevant miRNA was overexpressed in DU145 or PNT1B.** The relative quantification of these four proteins was measured by densitometry.
(DOC)

Figure S9 **The expression of p110α, p110δ, p85 and Akt increased after either PTEN inhibitor VO-OHpic trihydrate or PTEN siRNA#2 was imposed in DU145 or PNT1B.** (A) The mRNA expression of these four genes increased (mRNA / actin mRNA) after DU145 or PNT1B cells were treated with the PTEN inhibitor. (B) The protein expression of these four genes increased after DU145 or PNT1B cells were treated with PTEN inhibitor. (C) The mRNA expression of these four genes increased (mRNA / actin mRNA) after PTEN siRNA#2 was imposed in DU145 or PNT1B. (D) The protein expression of these four genes increased after PTEN siRNA#2 was imposed in DU145 or PNT1B. The relative quantification of these four proteins was measured by densitometry.
(DOC)

Figure S10 **The protein expression level of p110α, p110δ, p85 and Akt was altered after the relevant miRNA was neutralized in DU145 or PNT1B.** The relative quantification of these four proteins was measured by densitometry.
(DOC)

Figure S11 **Cyclin D1 was co-regulated by miR-19b, miR-23b and miR-92a at the post-transcriptional level.** (A) Prediction diagram of miRNA-binding site in the CCND1 (cyclin D1) mRNA 3’ UTR. There exsit three miR-19b binding sites, a miR-23b and a miR-92a binding site in its 3’ UTR. (B) Cyclin D1 was overexpressed in prostate cancer cell line DU145 compared with the PNT1B control. The relative quantification of cyclin D1 was measured by densitometry.
(DOC)

Figure S12 **Cyclin D1 expression increased upon treatment with PTEN inhibitor or siRNA interference in prostate cells.** Cyclin D1 expression increased upon treatment for DU145 (A) and PNT1B (B) cells with PTEN inhibitor or PTEN siRNA#2. The relative quantification of cyclin D1 was measured by densitometry.
(DOC)

Figure S13 **Overexpression of miR-19b, miR-23b, miR-26a or miR-92a stimulated cell proliferation of DU145 cells.** Cell growth was observed by daily counting for one week. Microphotographs of the cells were taken on day 4 after the cells were seeded. Original magnification: 100×.
(DOC)

Figure S14 **Cell growth was promoted after PTEN inhibitor or PTEN siRNA#2 was added to DU145 cells.** Cell growth was promoted after imposing PTEN inhibitor (A) or PTEN siRNA#2 (B) in DU145 cells. Cell growth was observed by daily counting for one week. Microphotographs of the cells were taken on day 4 after the cells were seeded. Original magnification: 100×. *indicates a significant difference from the control (p < 0.01).
(DOC)

Figure S15 **Overexpression of miR-19b, miR-23b, miR-26a or miR-92a stimulated the cell proliferation of PNT1B cells.** Cell growth was observed by daily counting for one week. Microphotographs of the cells were taken on day 4 after the cells were seeded. Original magnification: 100×.
(DOC)

Figure S16 **Cell growth was promoted after PTEN inhibitor or PTEN siRNA#2 was added to PNT1B cells.** Cell growth was promoted after imposing PTEN inhibitor (A) or PTEN siRNA#2 (B) in PNT1B cells. Cell growth was observed by daily counting for one week. Microphotographs of the cells were taken on day 4 after the cells were seeded. Original magnification: 100×. *indicates a significant difference from the control (p < 0.01).
(DOC)

Figure S17 **Overexpression of miR-26a alone or combination with miR-19b, miR-23b, or miR-92a stimulated cell proliferation in prostate cells.** MiR-26a alone or combination with miR-19b, miR-23b, or miR-92a was overexpressed in DU145 cells (A) or PNT1B cells (B). Cell growth was observed by daily counting for one week. Microphotographs of the cells were taken on day 4 after the cells were seeded. Original magnification: 100×. *indicates a significant difference from the control (p < 0.01).
(DOC)
