# Supplementary material for: Four MicroRNAs Promote Prostate Cell Proliferation with Regulation of PTEN and Its Downstream Signals In Vitro
Source: PLoS One. 2013 Sep 30;8(9):e75885. doi: 10.1371/journal.pone.0075885 (PMC3787937; doi:10.1371/journal.pone.0075885)
Supplement: Table S1 — Primers for amplifying truncated PTEN 3’ UTR fragments and their mutant counterparts. (DOC) [file pone.0075885.s001.doc]

**Table S1.** Primers for amplifying truncated PTEN 3’UTR fragments and their mutant counterparts.

| **Fragment** | **Forward** | **Reverse** |
| --- | --- | --- |
| PTEN 3’UTR part A  (PUA, mPUA) | 5’-GTTGCTAGCCTGATCCAGAGAATGAACCT-3’ | 5’-CACGATATCGAAACCTCTCTTAGCCAACT-3’ |
| PTEN 3’UTR part B  (PUB, mPUB) | 5’-GTTGCTAGCTTCCACCCTTTTGACCTTAC-3’ | 5’-CACGATATCGACCACAGCTAGTGAACAAT-3’ |
| PTEN 3’UTR part C  (PUC, mPUC) | 5’-GTAGCTAGCAATATGTAACATGGAGGGCC-3’ | 5’-CACGATATCTTCAAGAGGAGCTACAAAGG-3’ |
| PTEN 3’UTR part D  (PUD, mPUD) | 5’-GTTGCTAGCACTTAAGTGGAGTTTACCGG-3’ | 5’-CTCGATATCCCCACACAATGACAAGAATG-3’ |
