# Supplementary material for: Four MicroRNAs Promote Prostate Cell Proliferation with Regulation of PTEN and Its Downstream Signals In Vitro
Source: PLoS One. 2013 Sep 30;8(9):e75885. doi: 10.1371/journal.pone.0075885 (PMC3787937; doi:10.1371/journal.pone.0075885)
Supplement: Table S2 — Primers for amplifying pri-miRNA fragments. (DOC) [file pone.0075885.s002.doc]

**Table S2. Primers for amplifying pri-miRNA fragments.**

| **Fragment** | **Forward** | **Reverse** |
| --- | --- | --- |
| pri-miR-19b | 5’-GAGGATCCGTACTGCTAGCTGTAGAACTCC-3’ | 5’-GCTCTAGAGGACAAGTGCAATACCATACAG-3’ |
| pri-miR-23b | 5’-TTGGATCCAGCTACGAGATGCACCTGTT-3’ | 5’-GGTCTAGACTAGCATAACAGGCGTGAAG-3’ |
| pri-miR-26a | 5’-TTGGATCCGTCAGAAATTCTCTCCCGAGG-3’ | 5’-GGTCTAGATGTGAACTCTGGTGTTGGTGC-3’ |
| pri-miR-92a | 5’-GTGGATCCCTGTGTGATATTCTGCTGTGC-3’ | 5’-GGTCTAGATGCCAAATCTGACACGCAAC-3’ |
