# Supplementary material for: Four MicroRNAs Promote Prostate Cell Proliferation with Regulation of PTEN and Its Downstream Signals In Vitro
Source: PLoS One. 2013 Sep 30;8(9):e75885. doi: 10.1371/journal.pone.0075885 (PMC3787937; doi:10.1371/journal.pone.0075885)
Supplement: Table S3 — Primers for qRT-PCR. (DOC) [file pone.0075885.s003.doc]

**Table S3. Primers for qRT-PCR.**

| **Gene** | **Forward** | **Reverse** |
| --- | --- | --- |
| PTEN | 5’-TAGAGCGTGCAGATAATGAC-3’ | 5’-GGCTCCTCTACTGTTTTTGT-3’ |
| p110α | 5’-CAACAGCCACACACTACATC-3’ | 5’-TGTGACGATCTCCAATTCCC-3’ |
| p110β | 5’-GGGATTGGTGACAGACATAG-3’ | 5’-GAATAAAAGGCACTCGCTCC-3’ |
| p110δ | 5’-TCGCCAACATCCAACTCAAC-3’ | 5’-CACACAATAGCCAGCACAGG-3’ |
| p85 | 5’-CGGCGAAGTAAAGCATTGTG-3’ | 5’-ACATTGAGGGAGTCGTTGTG-3’ |
| Akt | 5’-CTTCTTTGCCGGTATCGTGT-3’ | 5’-TGTCATCTTGGTCAGGTGGT-3’ |
| Cyclin D1 | 5’-GTGCCACAGATGTGAAGTTC-3’ | 5’-CACACTTGATCACTCTGGAG-3’ |
| beta-actin | 5’-CATCCTCACCCTGAAGTACCC-3’ | 5’-AGCCTGGATAGCAACGTACATG-3’ |
