# Supplementary material for: Four MicroRNAs Promote Prostate Cell Proliferation with Regulation of PTEN and Its Downstream Signals In Vitro
Source: PLoS One. 2013 Sep 30;8(9):e75885. doi: 10.1371/journal.pone.0075885 (PMC3787937; doi:10.1371/journal.pone.0075885)
Supplement: Table S4 — Target sites of the four miRNAs in the 3’UTR of the key components of PI3K/Akt pathway. (DOC) [file pone.0075885.s021.doc]

**Table S4.** Target sites of the four miRNAs in the 3’UTR of the key components of PI3K/Akt pathway.

| **Gene** | **GenBank** | **3’UTR position in mRNA** | **miRNA** | **Binding sites in 3’UTR** | **Target Site** | |
| --- | --- | --- | --- | --- | --- | --- |
| PIK3CA (p110α) | NM_006218.2 | 3365-3710 | miR-19b | 3437-3459 | 3’-AGUCAAAACGU-ACCUAAACGUGU-5’  | | | || | | | | | ||| | | | |  5’-ACCG-ATTGCATAGGAA**TTGCAC**A-3’ | (miR-19b)  (3’UTR) |
| PIK3CD (p110δ) | NM_005026.3 | 3344-5411 | miR-26a | 3479-3501 | 3’-UCGGAUAGG-ACCUAAUGAACUU-5’  | | | | | | | | | | | | | |  5’-TGGTTATTTATTTATG**ACTTGA**A-3’ | (miR-26a)  (3’UTR) |
|  |  |  | miR-92a | 4147-4168 | 3’-UGUCCGGC-CCUGUUCACGUUAU-5’  ¦ \| \| \| \| \| ¦ \| \| \| \| \| \| \|  5’-GCTGG-AGTGCAGTGG**TGCAAT**C-3’ | (miR-92a)  (3’UTR) |
| PIK3R1 (p85) | NM_181523.2 | 2756-6975 | miR-23b | 5703-5724 | 3’- CCAUUAGGGACCGUUACACUA-5’  | ¦ | | | | | | | | | | |  5’-GAGAGAT-CATAACC**ATGTGA**A-3’ | (miR-23b)  (3’UTR) |
|  |  |  |  | 6086-6106 | 3’- CCAUUAGGGACCGUUACACUA-5’  \| \| \| \| \| \| \| \| \| \|  5’-GTCAAAATG-TGTTT**ATGTGA**G-3’ | (miR-23b)  (3’UTR) |
|  |  |  | miR-26a | 4985-5006 | 3’-UCGG-AU-AGGACCUAAUGAACUU-5’  \| \| \| \| \| \| \| \| \| \| \| \| \|  5’-ACTGTTAGCCCTCAA-**ACTTGA**C-3’ | (miR-26a)  (3’UTR) |
|  |  |  |  | 5378-5400 | 3’-UCG—GAUAGGACCUAAUGAACUU-5’  \| \| \| \| \| \| \| \| \| \| \| \| \|  5’-TTCATCATTCC—ATATG**ACTTGA**G-3’ | (miR-26a)  (3’UTR) |
|  |  |  | miR-92a | 3547-3568 | 3’-UGUCCG-GCCCUGUUCACGUUAU-5’  \| \| \| \| ¦ \| \| \| \| \| \| \| \|  5’-AAATTCACAGCGCTA-**TGCAAT**T-3’ | (miR-92a)  (3’UTR) |
|  |  |  |  | 4752-4776 | 3’-UGUCCGG—CCCUGUU-CACGUUAU-5’  \| \| \| \| \| \| \| \| \| \| \| \| \|  5’-ATAGACTAAATGAAATCT**TGCAAT**T-3’ | (miR-92a)  (3’UTR) |
|  |  |  |  | 5328-5349 | 3’-UGUCCGGCCCUGUUCACGUUAU-5’  \| \| \| \| \| \| \| \| \| \| \| \|  5’-TCTCACAAACATAAG**TGCAAT**A-3’ | (miR-92a)  (3’UTR) |
| AKT | NM_005163.2 | 1998-2990 | miR-26a | 2750-2774 | 3’-UCG-GAUAGG-ACC-UAAUGAACUU-5’  | | | | | | | | | | | | | |  5’-TGATCTCTCCACGGTAGC**ACTTGA**C-3’ | (miR-26a)  (3’UTR) |
