# Supplementary material for: Four MicroRNAs Promote Prostate Cell Proliferation with Regulation of PTEN and Its Downstream Signals In Vitro
Source: PLoS One. 2013 Sep 30;8(9):e75885. doi: 10.1371/journal.pone.0075885 (PMC3787937; doi:10.1371/journal.pone.0075885)
Supplement: Figure S1 — Sequences of RNA-target sites for the four miRNAs in the PTEN 3’ UTR. Sequences of RNA-target sites for miR-19b (A), miR-23b (B), miR-26a (C) and miR-92a (D) in the PTEN 3’ UTR are shown paired with specific miRNA sequences. The seed region of each miRNA is underlined. (DOC) [file pone.0075885.s004.doc]

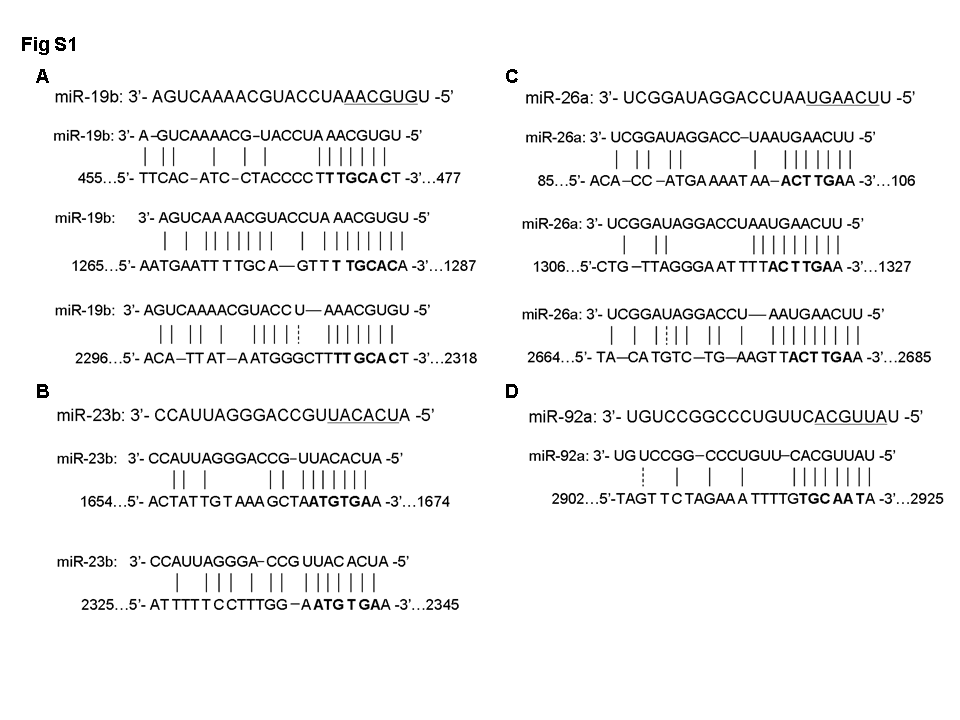


**Figure S1.** Sequences of RNA-target sites for the four miRNAs in the PTEN 3’UTR. Sequences of RNA-target sites for miR-19b (A), miR-23b (B), miR-26a (C) and miR-92a (D) in the PTEN 3’UTR are shown paired with specific miRNA sequences. The seed region of each miRNA is underlined.
