# Supplementary material for: Four MicroRNAs Promote Prostate Cell Proliferation with Regulation of PTEN and Its Downstream Signals In Vitro
Source: PLoS One. 2013 Sep 30;8(9):e75885. doi: 10.1371/journal.pone.0075885 (PMC3787937; doi:10.1371/journal.pone.0075885)
Supplement: Figure S2 — Validation of miRNA targets in the PTEN 3’ UTR. (A) PNT1B cells were co-transfected the luciferase reporter vectors, which harbored either miR-target positive control (PC), full-length PTEN 3’ UTR (PU), or its mutant counterpart (mPU) respectively, with relevant anti-miRNA inhibitors for the luciferase reporter assay. (B-E) Luciferase reporter vectors containing relevant truncated fragment PUA (B), PUB (C), PUC (D) or PUD (E) of PTEN 3’ UTR with (MT) or without (WT) mutant(s) in its relevant miRNA binding site(s) were co-transfected with specific anti-miRNA inhibitors respectively for the luciferase reporter assay. *indicates a significant difference from the control (p<0.01). (DOC) [file pone.0075885.s005.doc]

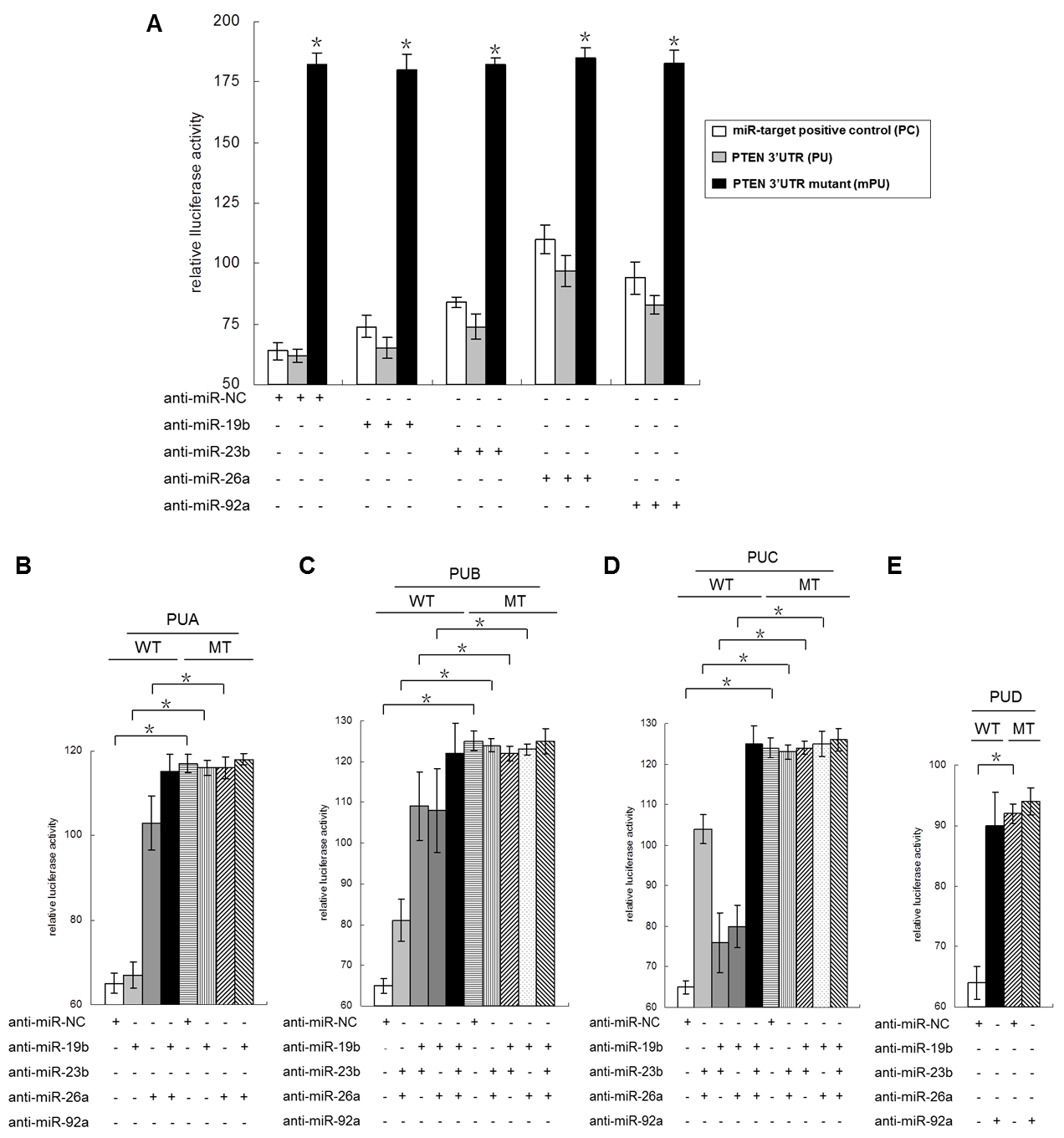


**Figure S2.** Validation of miRNA targets in the PTEN 3’UTR. (A) PNT1B cells were co-transfected the luciferase reporter vectors, which harbored either miR-target positive control (PC), full-length PTEN 3’UTR (PU), or its mutant counterpart (mPU) respectively, with relevant anti-miRNA inhibitors for the luciferase reporter assay. (B-E) Luciferase reporter vectors containing relevant truncated fragment PUA (B), PUB (C), PUC (D) or PUD (E) of PTEN 3’UTR with (MT) or without (WT) mutant(s) in its relevant miRNA binding site(s) were co-transfected with specific anti-miRNA inhibitors respectively for the luciferase reporter assay. ＊indicates a significant difference from the control (p<0.01).
