# Supplementary material for: Four MicroRNAs Promote Prostate Cell Proliferation with Regulation of PTEN and Its Downstream Signals In Vitro
Source: PLoS One. 2013 Sep 30;8(9):e75885. doi: 10.1371/journal.pone.0075885 (PMC3787937; doi:10.1371/journal.pone.0075885)
Supplement: Figure S3 — TaqMan microRNA assay for the specific miRNA expression in DU145. (A) The four miRNAs were overexpressed after transient transfection of the specific miRNA expression vectors. (B) The four miRNAs were neutralized after the specific anti-miRNA inhibitors were imposed. (DOC) [file pone.0075885.s006.doc]

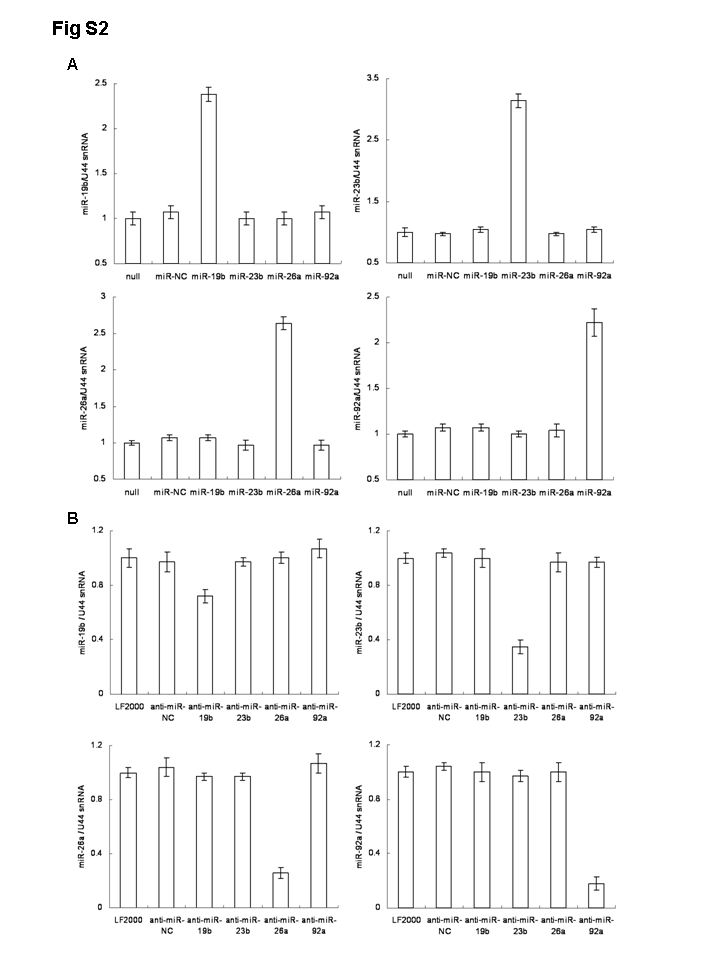


**Figure S3.** TaqMan microRNA assay for the specific miRNA expression in DU145. (A) The four miRNAs were overexpressed after transient transfection of the specific miRNA expression vectors. (B) The four miRNAs were neutralized after the specific anti-miRNA inhibitors were imposed.
