# Supplementary material for: Four MicroRNAs Promote Prostate Cell Proliferation with Regulation of PTEN and Its Downstream Signals In Vitro
Source: PLoS One. 2013 Sep 30;8(9):e75885. doi: 10.1371/journal.pone.0075885 (PMC3787937; doi:10.1371/journal.pone.0075885)
Supplement: Figure S5 — Up-regulation of PTEN expression by neutralizing the specific miRNAs in PNT1B. The expression of PTEN was up-regulated after two (A) or three (B) of four miRNAs were neutralized simultaneously in PNT1B. The relative quantification of PTEN protein was measured by densitometry. (DOC) [file pone.0075885.s008.doc]

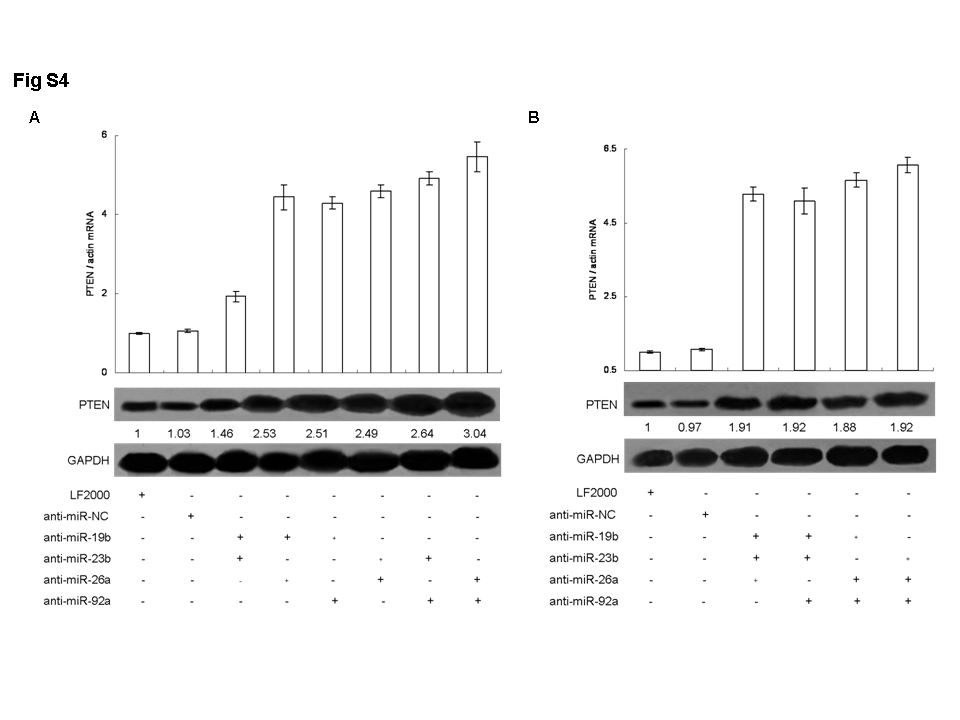


**Figure S5.** Up-regulation of PTEN expression by neutralizing the specific miRNAs in PNT1B. The expression of PTEN was up-regulated after two (A) or three (B) of the four miRNAs were neutralized simultaneously in PNT1B. The relative quantification of PTEN protein was measured by densitometry.
