# Supplementary material for: Four MicroRNAs Promote Prostate Cell Proliferation with Regulation of PTEN and Its Downstream Signals In Vitro
Source: PLoS One. 2013 Sep 30;8(9):e75885. doi: 10.1371/journal.pone.0075885 (PMC3787937; doi:10.1371/journal.pone.0075885)
Supplement: Figure S6 — Repression of PTEN by PTEN-specific siRNA interference. PTEN expression was repressed by PTEN-specific siRNAs in DU145 (A) and PNT1B (B), and among them PTEN siRNA#2 was identified as the most effective silencer for the following experiments and to be used as a positive control. The relative quantification of PTEN protein was measured by densitometry. (DOC) [file pone.0075885.s009.doc]

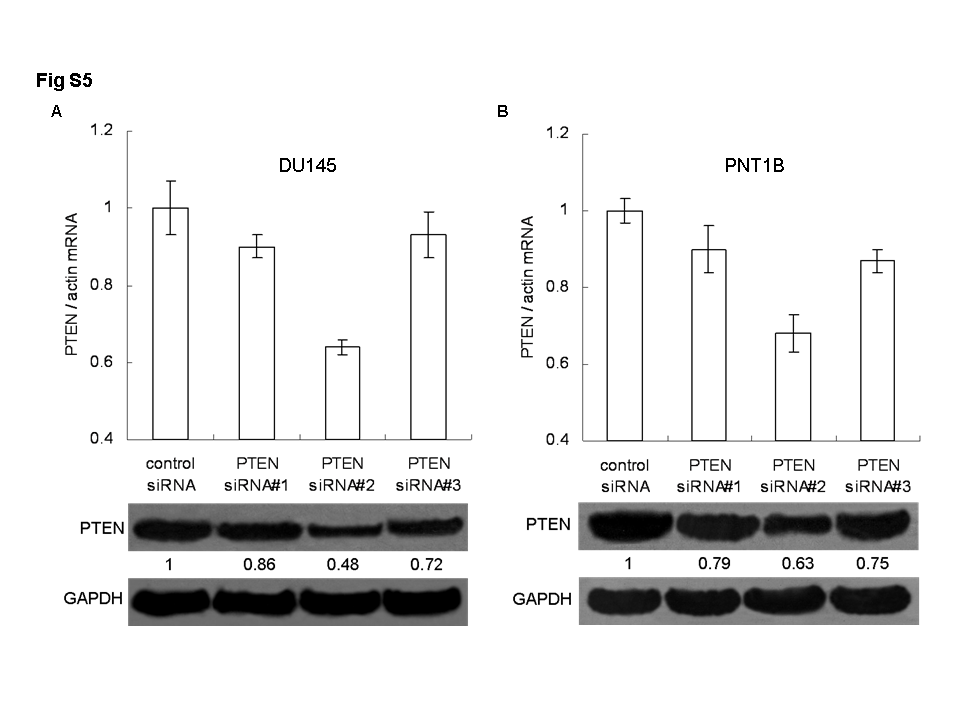


**Figure S6.** Repression of PTEN by PTEN-specific siRNA interference. PTEN expression was repressed by PTEN-specific siRNAs in DU145 (A) and PNT1B (B), and among them PTEN siRNA#2 was identified as the most effective silencer for the following experiments and to be used as a positive control. The relative quantification of PTEN protein was measured by densitometry.
