# Supplementary material for: Four MicroRNAs Promote Prostate Cell Proliferation with Regulation of PTEN and Its Downstream Signals In Vitro
Source: PLoS One. 2013 Sep 30;8(9):e75885. doi: 10.1371/journal.pone.0075885 (PMC3787937; doi:10.1371/journal.pone.0075885)
Supplement: Figure S7 — Prediction diagram of miRNA targeting site in the PIK3CA (p110α), PIK3CD (p110δ), PIK3R1 (p85) and Akt mRNA 3’ UTR. PIK3CA 3’ UTR harbors a miR-19b targeting site, while PIK3CD 3’ UTR harbors a miR-26a and a miR-92a targeting site. In the PIK3R1 3’ UTR, multiple miR-23b, miR-26a and miR-92a targeting sites can be found. In Akt 3’ UTR, a miR-26a targeting site is located. (DOC) [file pone.0075885.s010.doc]

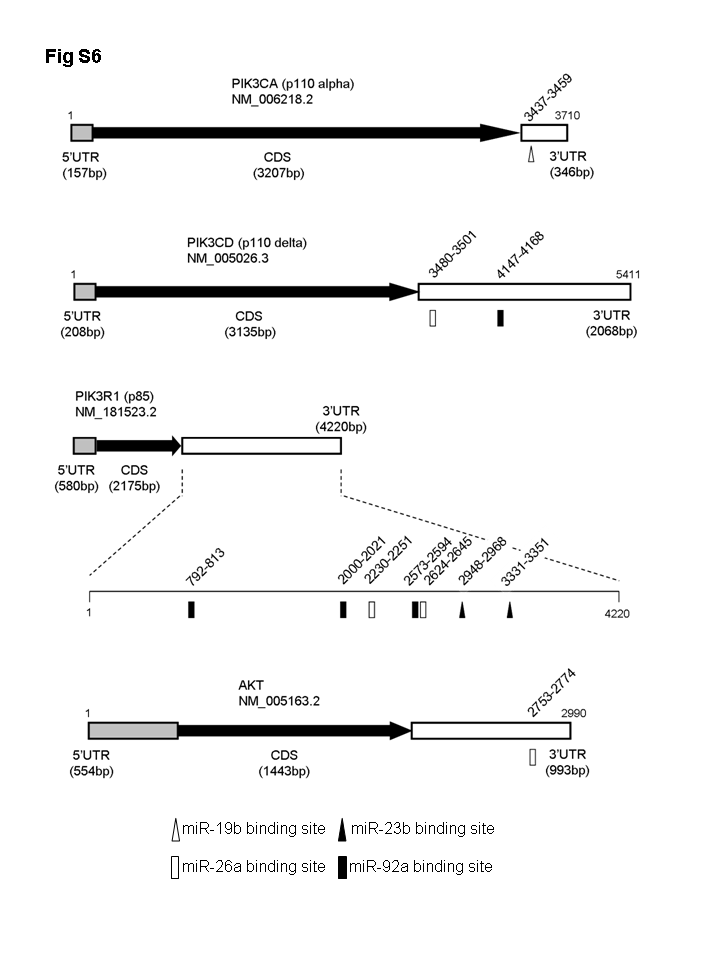


**Figure S7.** Prediction diagram of miRNA targeting site in the PIK3CA (p110α), PIK3CD (p110δ), PIK3R1 (p85) and Akt mRNA 3’UTR. PIK3CA 3’UTR harbors a miR-19b targeting site, while PIK3CD 3’UTR harbors a miR-26a and a miR-92a targeting site. In the PIK3R1 3’UTR, multiple miR-23b, miR-26a and miR-92a targeting sites can be found. In Akt 3’UTR, a miR-26a targeting site is located.
