# Supplementary material for: Four MicroRNAs Promote Prostate Cell Proliferation with Regulation of PTEN and Its Downstream Signals In Vitro
Source: PLoS One. 2013 Sep 30;8(9):e75885. doi: 10.1371/journal.pone.0075885 (PMC3787937; doi:10.1371/journal.pone.0075885)
Supplement: Figure S9 — The expression of p110α, p110δ, p85 and Akt increased after either PTEN inhibitor VO-OHpic trihydrate or PTEN siRNA#2 was imposed in DU145 or PNT1B. (A) The mRNA expression of these four genes increased (mRNA / actin mRNA) after DU145 or PNT1B cells were treated with the PTEN inhibitor. (B) The protein expression of these four genes increased after DU145 or PNT1B cells were treated with PTEN inhibitor. (C) The mRNA expression of these four genes increased (mRNA / actin mRNA) after PTEN siRNA#2 was imposed in DU145 or PNT1B. (D) The protein expression of these four genes increased after PTEN siRNA#2 was imposed in DU145 or PNT1B. The relative quantification of these four proteins was measured by densitometry. (DOC) [file pone.0075885.s012.doc]

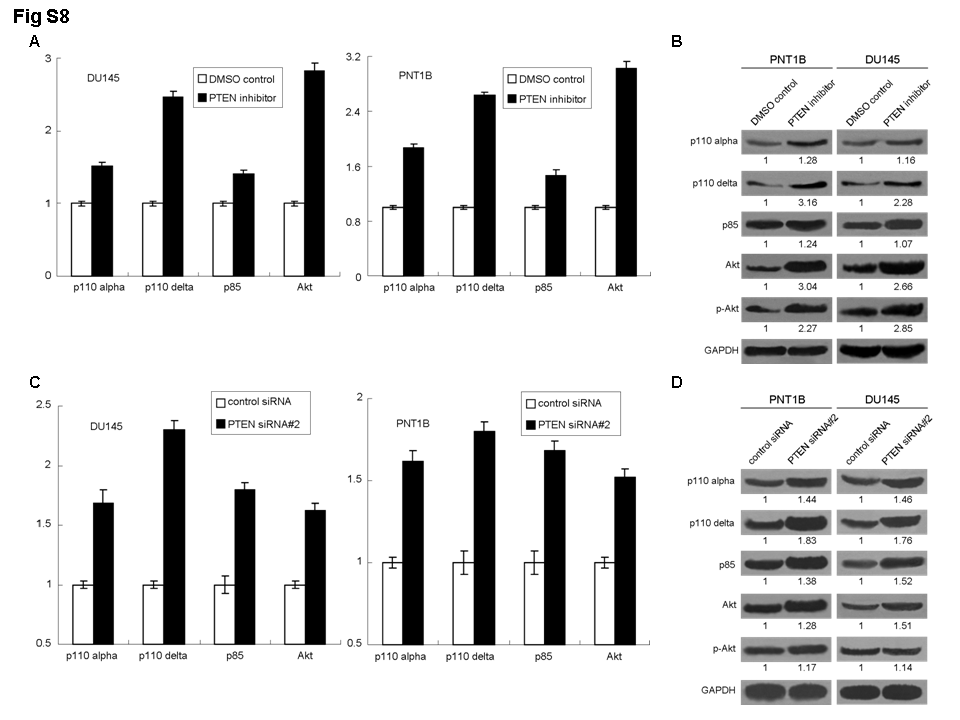


**Figure S9.** The expression of p110α, p110δ, p85 and Akt increased after either PTEN inhibitor VO-OHpic trihydrate or PTEN siRNA#2 was imposed in DU145 or PNT1B. (A) The mRNA expression of these four genes increased (mRNA / actin mRNA) after DU145 or PNT1B cells were treated with the PTEN inhibitor. (B) The protein expression of these four genes increased after DU145 or PNT1B cells were treated with PTEN inhibitor. (C) The mRNA expression of these four genes increased (mRNA / actin mRNA) after PTEN siRNA#2 was imposed in DU145 or PNT1B. (D) The protein expression of these four genes increased after PTEN siRNA#2 was imposed in DU145 or PNT1B. The relative quantification of these four proteins was measured by densitometry.
