# Supplementary material for: Four MicroRNAs Promote Prostate Cell Proliferation with Regulation of PTEN and Its Downstream Signals In Vitro
Source: PLoS One. 2013 Sep 30;8(9):e75885. doi: 10.1371/journal.pone.0075885 (PMC3787937; doi:10.1371/journal.pone.0075885)
Supplement: Figure S10 — The protein expression level of p110α, p110δ, p85 and Akt was altered after the relevant miRNA was neutralized in DU145 or PNT1B. The relative quantification of these four proteins was measured by densitometry. (DOC) [file pone.0075885.s013.doc]

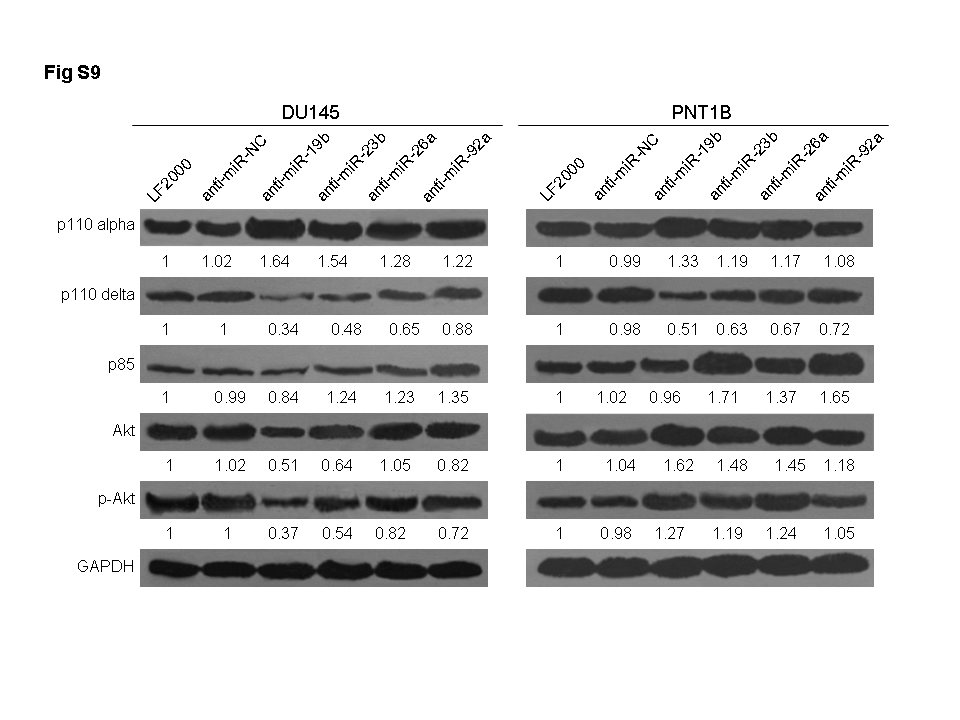


**Figure S10.** The protein expression level of p110α, p110δ, p85 and Akt was altered after the relevant miRNA was neutralized in DU145 or PNT1B. The relative quantification of these four proteins was measured by densitometry.
