# Supplementary material for: Four MicroRNAs Promote Prostate Cell Proliferation with Regulation of PTEN and Its Downstream Signals In Vitro
Source: PLoS One. 2013 Sep 30;8(9):e75885. doi: 10.1371/journal.pone.0075885 (PMC3787937; doi:10.1371/journal.pone.0075885)
Supplement: Figure S11 — Cyclin D1 was co-regulated by miR-19b, miR-23b and miR-92a at the post-transcriptional level. (A) Prediction diagram of miRNA-binding site in the CCND1 (cyclin D1) mRNA 3’ UTR. There exsit three miR-19b binding sites, a miR-23b and a miR-92a binding site in its 3’ UTR. (B) Cyclin D1 was overexpressed in prostate cancer cell line DU145 compared with the PNT1B control. The relative quantification of cyclin D1 was measured by densitometry. (DOC) [file pone.0075885.s014.doc]

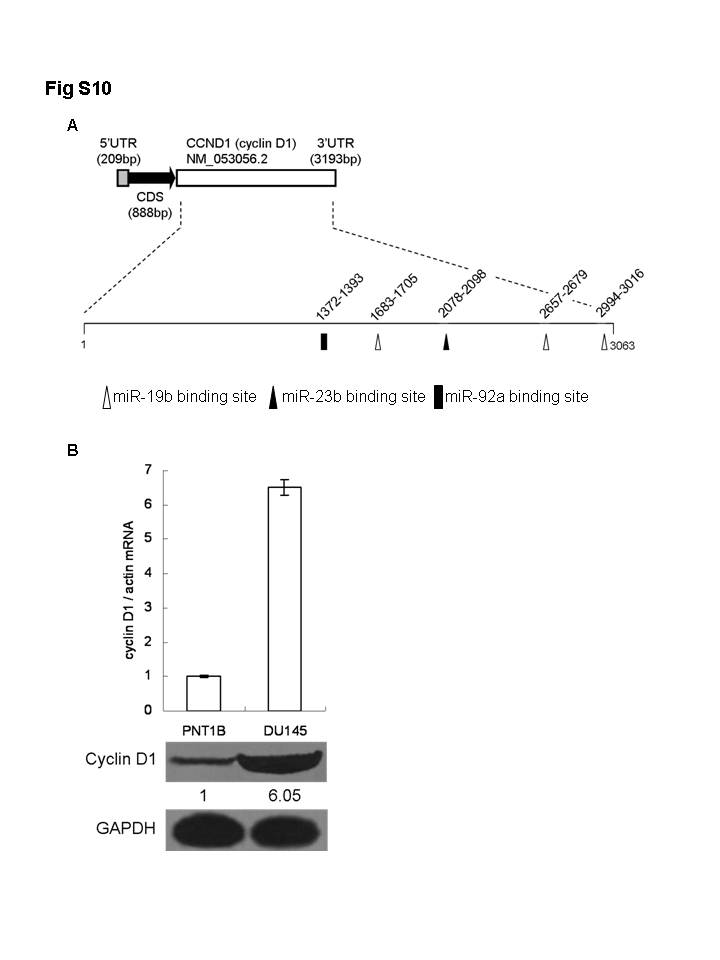


**Figure S11.** Cyclin D1 was co-regulated by miR-19b, miR-23b and miR-92a at the post-transcriptional level. (A) Prediction diagram of miRNA-binding site in the CCND1 (cyclin D1) mRNA 3’UTR. There exsit three miR-19b binding sites, a miR-23b and a miR-92a binding site in its 3’UTR. (B) Cyclin D1 was overexpressed in prostate cancer cell line DU145 compared with the PNT1B control. The relative quantification of cyclin D1 was measured by densitometry.
