# Supplementary material for: Four MicroRNAs Promote Prostate Cell Proliferation with Regulation of PTEN and Its Downstream Signals In Vitro
Source: PLoS One. 2013 Sep 30;8(9):e75885. doi: 10.1371/journal.pone.0075885 (PMC3787937; doi:10.1371/journal.pone.0075885)
Supplement: Figure S12 — Cyclin D1 expression increased upon treatment with PTEN inhibitor or siRNA interference in prostate cells. Cyclin D1 expression increased upon treatment for DU145 (A) and PNT1B (B) cells with PTEN inhibitor or PTEN siRNA#2. The relative quantification of cyclin D1 was measured by densitometry. (DOC) [file pone.0075885.s015.doc]

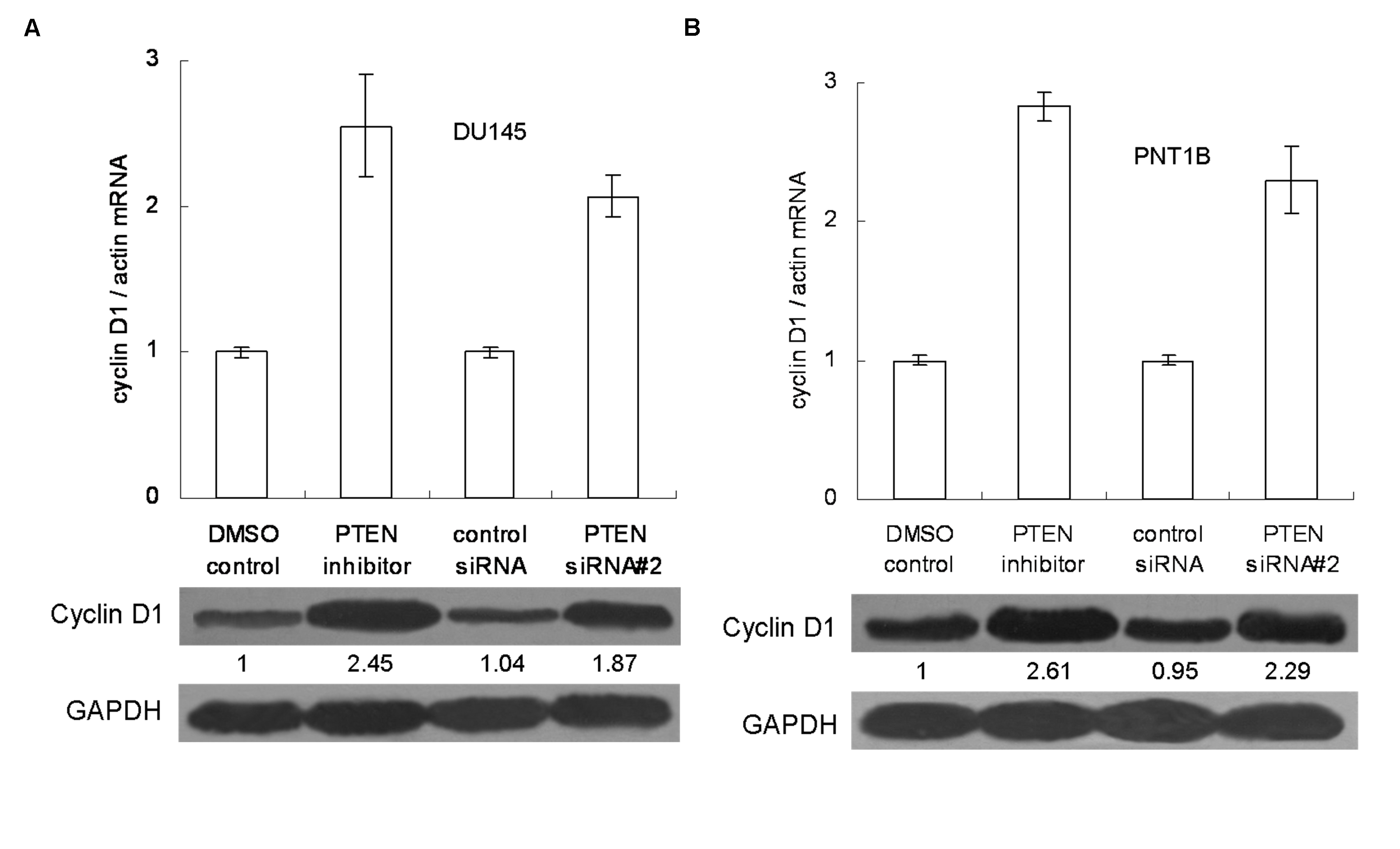


**Figure S12.** Cyclin D1 expression increased upon treatment with PTEN inhibitor or siRNA interference in prostate cells. Cyclin D1 expression increased upon treatment for DU145 (A) and PNT1B (B) cells with PTEN inhibitor or PTEN siRNA#2. The relative quantification of cyclin D1 was measured by densitometry.
