# Supplementary material for: Four MicroRNAs Promote Prostate Cell Proliferation with Regulation of PTEN and Its Downstream Signals In Vitro
Source: PLoS One. 2013 Sep 30;8(9):e75885. doi: 10.1371/journal.pone.0075885 (PMC3787937; doi:10.1371/journal.pone.0075885)
Supplement: Figure S13 — Overexpression of miR-19b, miR-23b, miR-26a or miR-92a stimulated cell proliferation of DU145 cells. Cell growth was observed by daily counting for one week. Microphotographs of the cells were taken on day 4 after the cells were seeded. Original magnification: 100×. (DOC) [file pone.0075885.s016.doc]

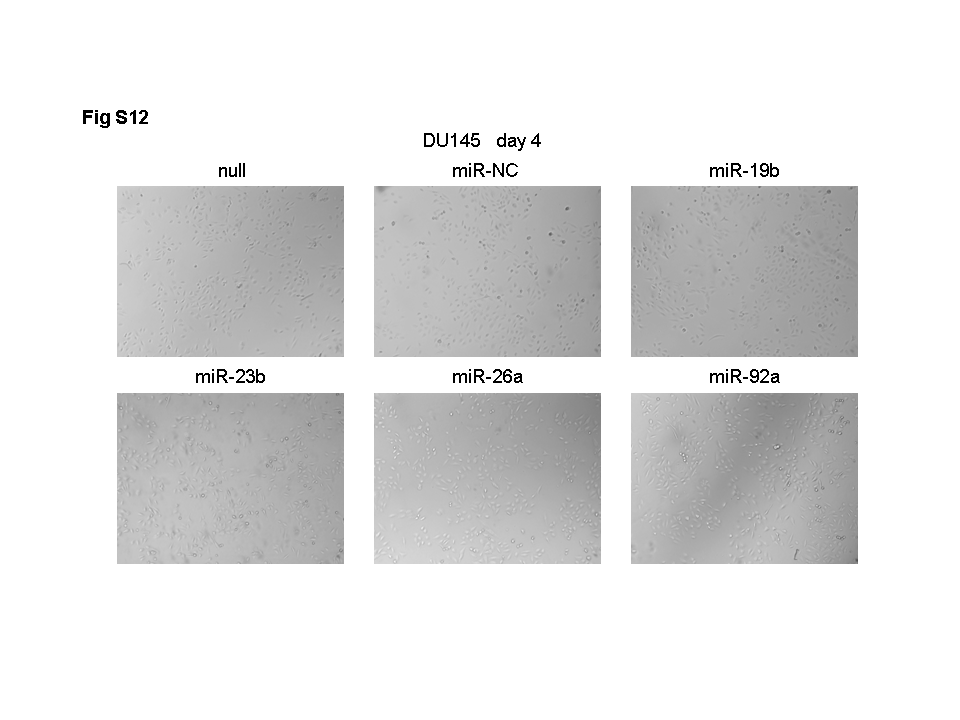


**Figure S13.** Overexpression of miR-19b, miR-23b, miR-26a or miR-92a stimulated cell proliferation of DU145 cells. Cell growth was observed by daily counting for one week. Microphotographs of the cells were taken on day 4 after the cells were seeded. Original magnification: 100×.
