# Supplementary material for: Four MicroRNAs Promote Prostate Cell Proliferation with Regulation of PTEN and Its Downstream Signals In Vitro
Source: PLoS One. 2013 Sep 30;8(9):e75885. doi: 10.1371/journal.pone.0075885 (PMC3787937; doi:10.1371/journal.pone.0075885)
Supplement: Figure S14 — Cell growth was promoted after PTEN inhibitor or PTEN siRNA#2 was added to DU145 cells. Cell growth was promoted after imposing PTEN inhibitor (A) or PTEN siRNA#2 (B) in DU145 cells. Cell growth was observed by daily counting for one week. Microphotographs of the cells were taken on day 4 after the cells were seeded. Original magnification: 100×. *indicates a significant difference from the control (p < 0.01). (DOC) [file pone.0075885.s017.doc]

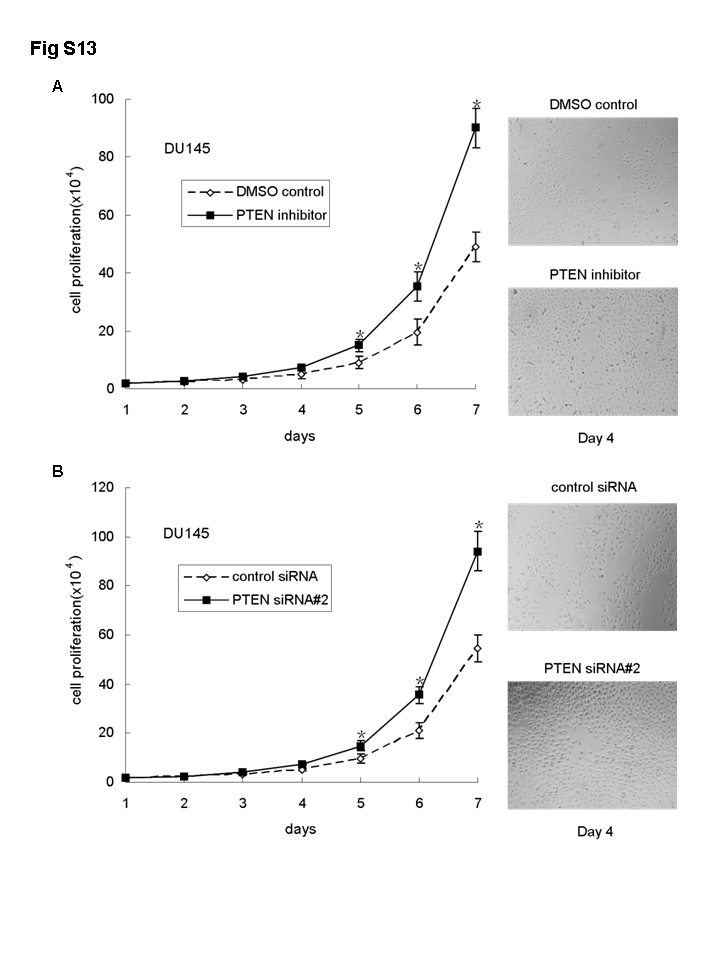


**Figure S14.** Cell growth was promoted after PTEN inhibitor or PTEN siRNA#2 was added to DU145 cells. Cell growth was promoted after imposing PTEN inhibitor (A) or PTEN siRNA#2 (B) in DU145 cells. Cell growth was observed by daily counting for one week. Microphotographs of the cells were taken on day 4 after the cells were seeded. Original magnification: 100×. ＊indicates a significant difference from the control (p < 0.01).
