# Supplementary material for: Four MicroRNAs Promote Prostate Cell Proliferation with Regulation of PTEN and Its Downstream Signals In Vitro
Source: PLoS One. 2013 Sep 30;8(9):e75885. doi: 10.1371/journal.pone.0075885 (PMC3787937; doi:10.1371/journal.pone.0075885)
Supplement: Figure S17 — Overexpression of miR-26a alone or combination with miR-19b, miR-23b, or miR-92a stimulated cell proliferation in prostate cells. MiR-26a alone or combination with miR-19b, miR-23b, or miR-92a was overexpressed in DU145 cells (A) or PNT1B cells (B). Cell growth was observed by daily counting for one week. Microphotographs of the cells were taken on day 4 after the cells were seeded. Original magnification: 100×. *indicates a significant difference from the control (p < 0.01). (DOC) [file pone.0075885.s020.doc]

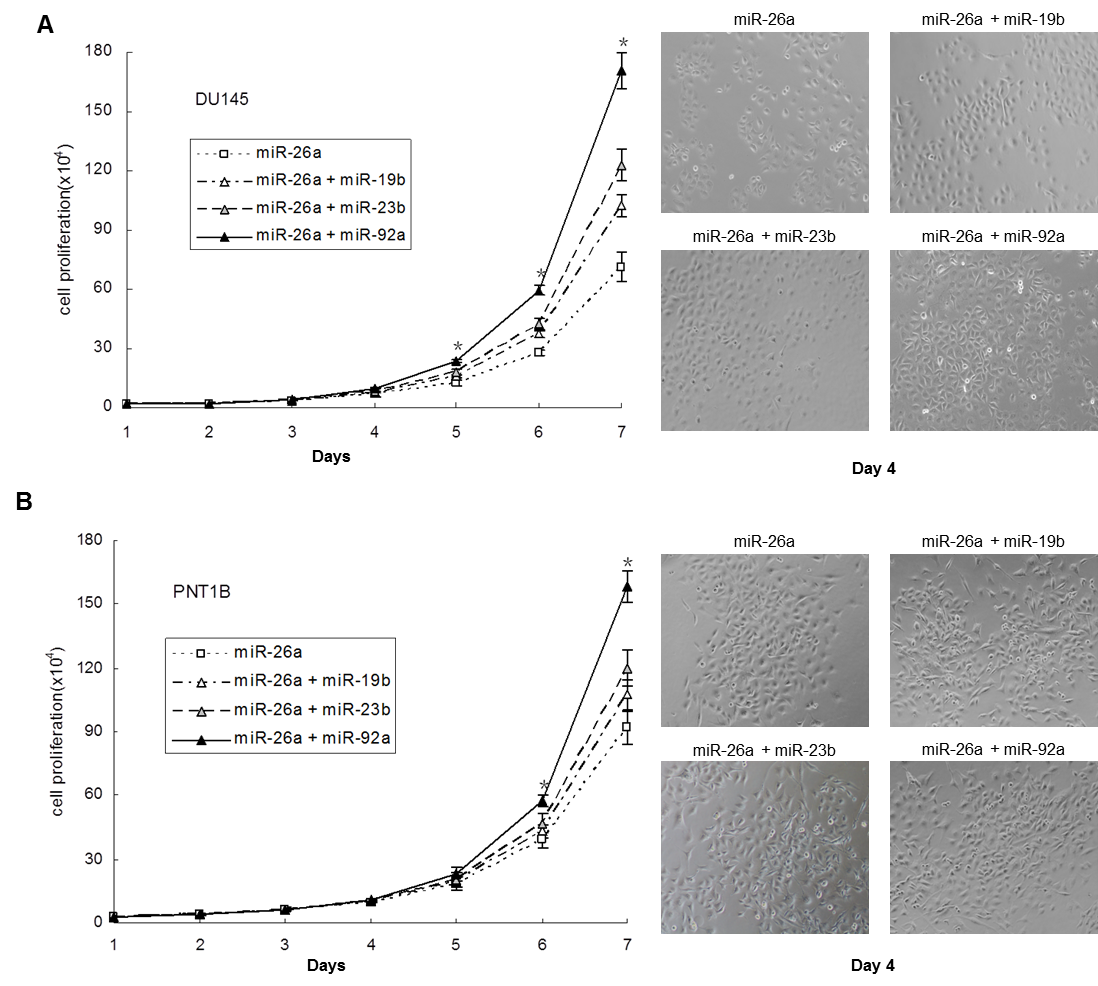


**Figure S17.** Overexpression of miR-26a alone or combination with miR-19b, miR-23b, or miR-92a stimulated cell proliferation in prostate cells. miR-26a alone or combination with miR-19b, miR-23b, or miR-92a was overexpressed in DU145 cells (A) or PNT1B cells (B). Cell growth was observed by daily counting for one week. Microphotographs of the cells were taken on day 4 after the cells were seeded. Original magnification: 100×. ＊indicates a significant difference from the control (p < 0.01).
